# Supplementary material for: Simultaneous inhibition of DNA-PK and Polϴ improves integration efficiency and precision of genome editing
Source: Nat Commun. 2023 Aug 14;14:4761. doi: 10.1038/s41467-023-40344-4 (PMC10425386; doi:10.1038/s41467-023-40344-4)
Supplement: Supplementary file 1 — Supplementary Information [file 41467_2023_40344_MOESM1_ESM.docx]

**Simultaneous inhibition of DNA-PK and Polϴ improves integration efficiency and precision of genome editing**

**Supplementary Figures S1-12**

**Supplementary Note 1 & 2**

**Supplementary Figure S1**

**
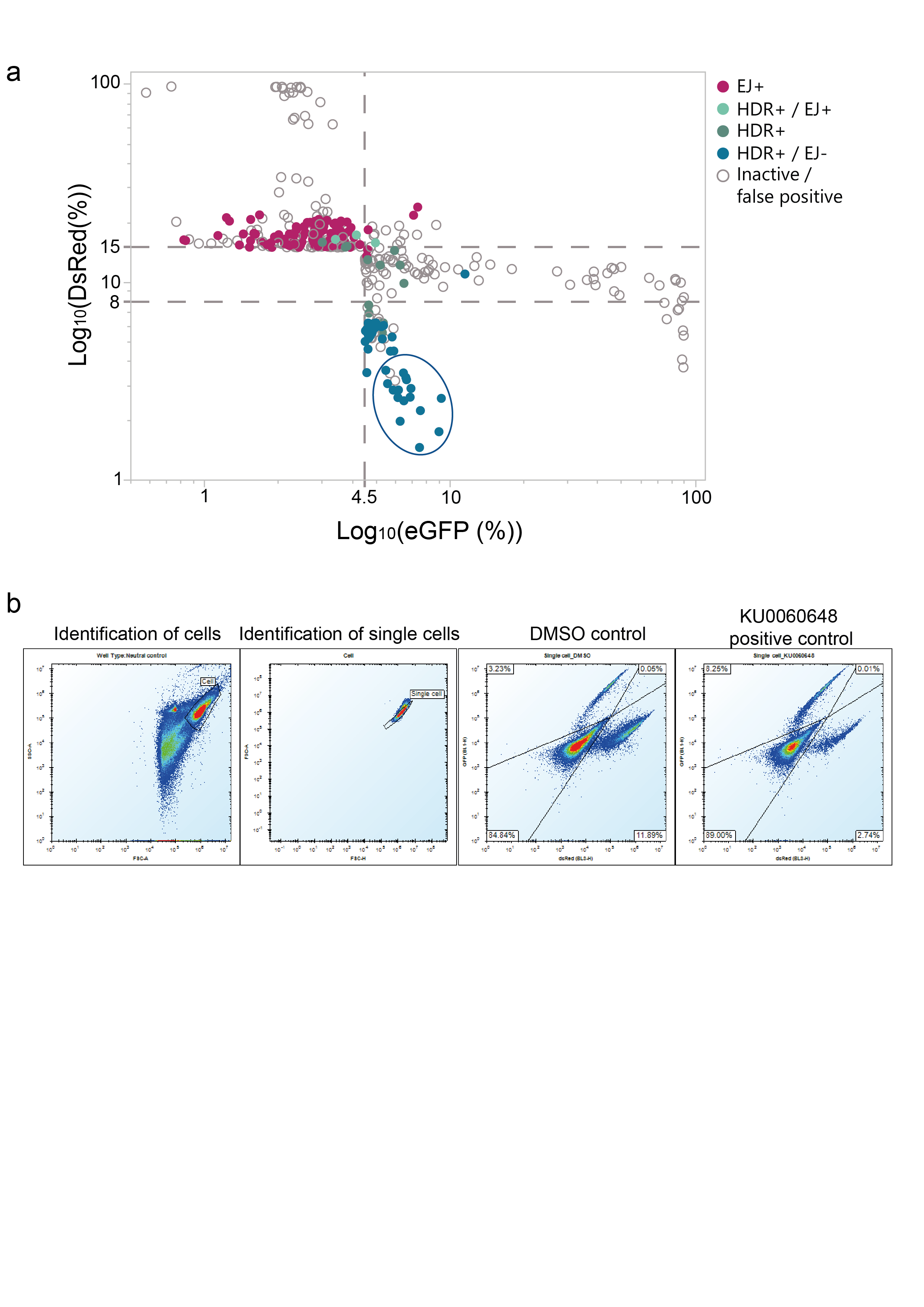
**

**Supplementary Figure S1 related to Figure 1: Validation of primary high-throughput screening results by a hit confirmation screen.** (**a**) Dots represent revised categories of the primary screen values by the outcome of the hit confirmation screen (n=1). (**b**) Representative flow cytometry plots of DMSO and KU0060648 treated cells. Gating strategy for cells based on forward scatter (FSC) and side scatter (SSC), subsequently gated for singlets with FSC height (FSC-H) and FSC-area (FSC-A), followed by eGFP and DsRed quantification in the single cell population. Source data are provided as a Source Data file.

**Supplementary Figure S2**

**
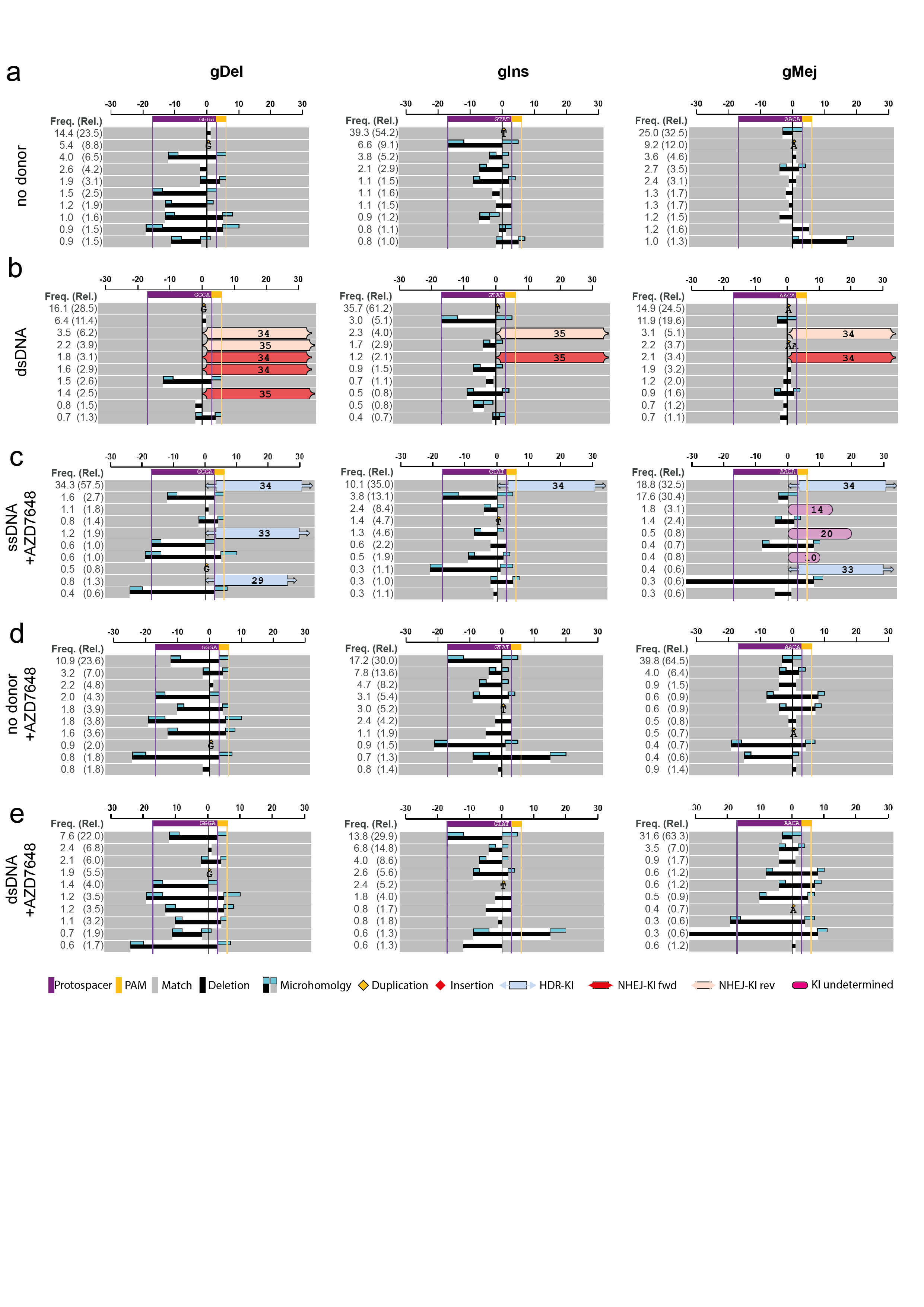
**

**Supplementary Figure S2 related to Figure 2: Graphical presentation of Knock-In Sequencing (KI-Seq) analysis to assess DNA repair outcomes at *Sp*Cas9-induced DNA Double-Strand Breaks (DSBs).** Representative InDel profiles +/-30 bp around the cut site from *Sp*Cas9 edited HEK293T cells at indicated loci (**a-d**) without DNA donor, (**b,d**) with double-stranded DNA (dsDNA) (**c**) or single-stranded DNA (ssDNA) donor. Cells were either treated with (**a,b**) DMSO or (**c-e**) 1 µM AZD7648. Each variant is annotated with absolute frequencies (Freq.) = fraction of mapped reads and relative frequencies (Rel.) in brackets = fraction of mutated reads. PAM: protospacer adjacent motif; HDR-KI: ssDNA donor with homology arms; NHEJ-KI: dsDNA donor without homology arms; KI undetermined: insertions >10 bp.

**Supplementary Figure S3**

**
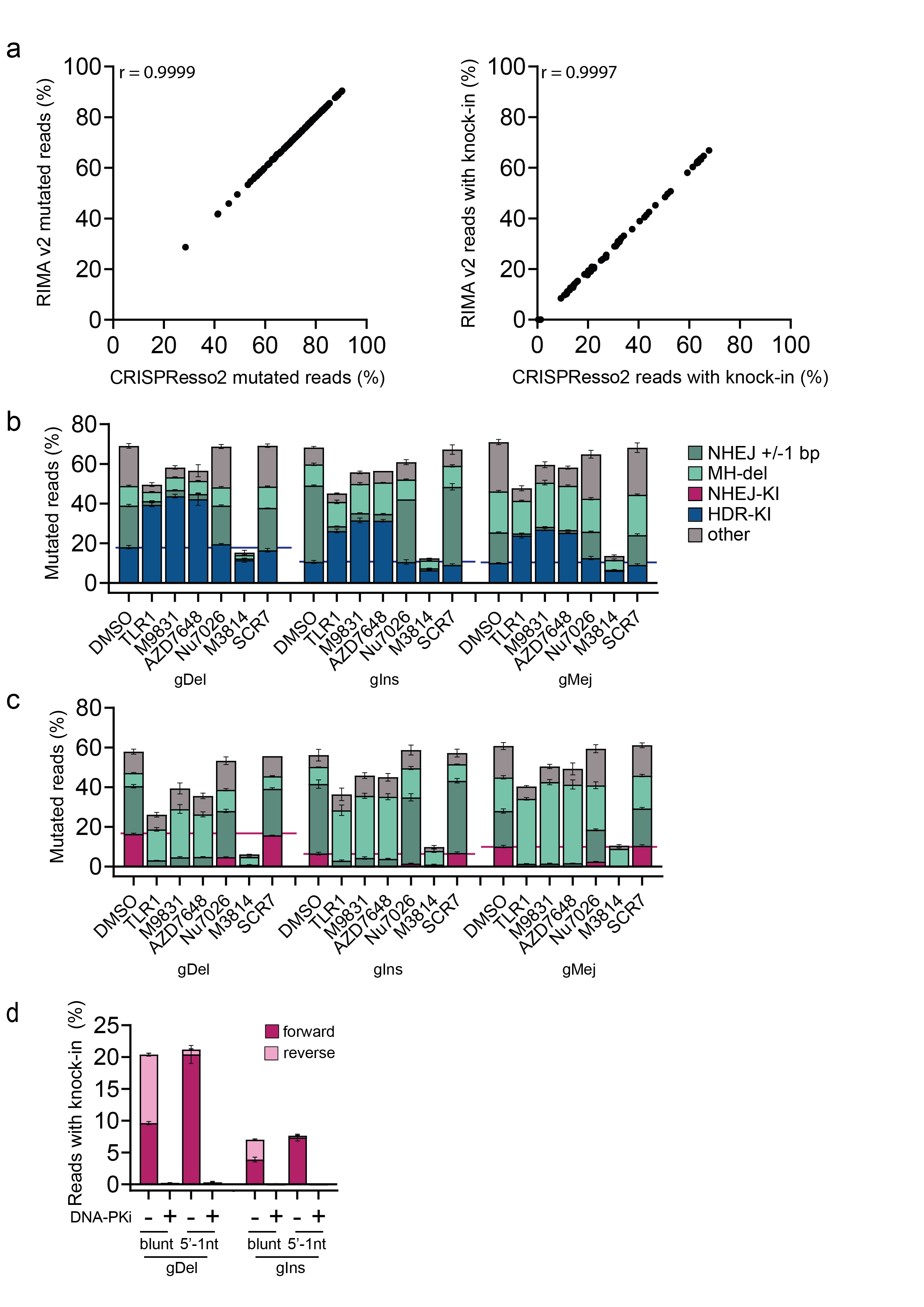
**

**Supplementary Figure S3 related to Figure 2: Knock-In Sequencing (KI-Seq) determines DNA repair outcomes at *Sp*Cas9-induced DNA Double-Strand Breaks (DSBs).** (**a**) Benchmarking KI-Seq to widely used CRISPResso2. Comparison of fraction of mutated reads and percentage of reads with knock-in (perfect + imperfect knock-ins) of samples from several experiments (n=137). The dataset contains samples from three target sites, with and without ssDNA, and treatment with 1 µM AZD7648 or corresponding DMSO controls. Pearson correlation coefficient is shown. (**b,c**) Impact of NHEJ-repair pathway inhibitors on editing frequencies at three target sites. Alterations to the reference DNA sequence are assigned to different DNA DSB repair pathways: +/-1 bp insertions/deletions = non-homologous end joining (NHEJ), deletions with microhomologies (MH-del), *i.e.,* ≥2 bp = alternative end joining (alt-EJ), integration of double-stranded DNA (dsDNA) donor without homology arms = NHEJ-KI, integration of a single-stranded DNA (ssDNA) donor with homology arms = HDR-KI, all remaining events are summarised in “other”. (**b**) In the presence of ssDNA or (**c**) dsDNA donor. DNA-PK inhibitors from the Traffic Light Reporter (TLR) screen TLR1 (3 µM), M9831 (3 µM) and AZD7648 (3 µM) are compared to previously published compounds targeting DNA-PK. Nu7026 (5.6 µM), M3814 (3 µM) or LIG4 SCR7 (10 µM). Bar graphs depict mean percentage of mutated reads ± standard deviation (n=3, technical replicates) in mapped reads. (**d**) *Sp*Cas9 promotes directional NHEJ-mediated KI. Bar graphs show mean forward and reverse insertion efficiencies ± standard deviation (n=3, technical replicates) for two types of dsDNA donor (blunt-ended and one nucleotide 5’-overhangs) at two target sites assessed with KI-Seq. Experiments were performed with 1 µM DNA-PK inhibitor (M9831), or DMSO control added 3 hours before plasmid transfections. Source data are provided as a Source Data file.

**Supplementary Figure S4**


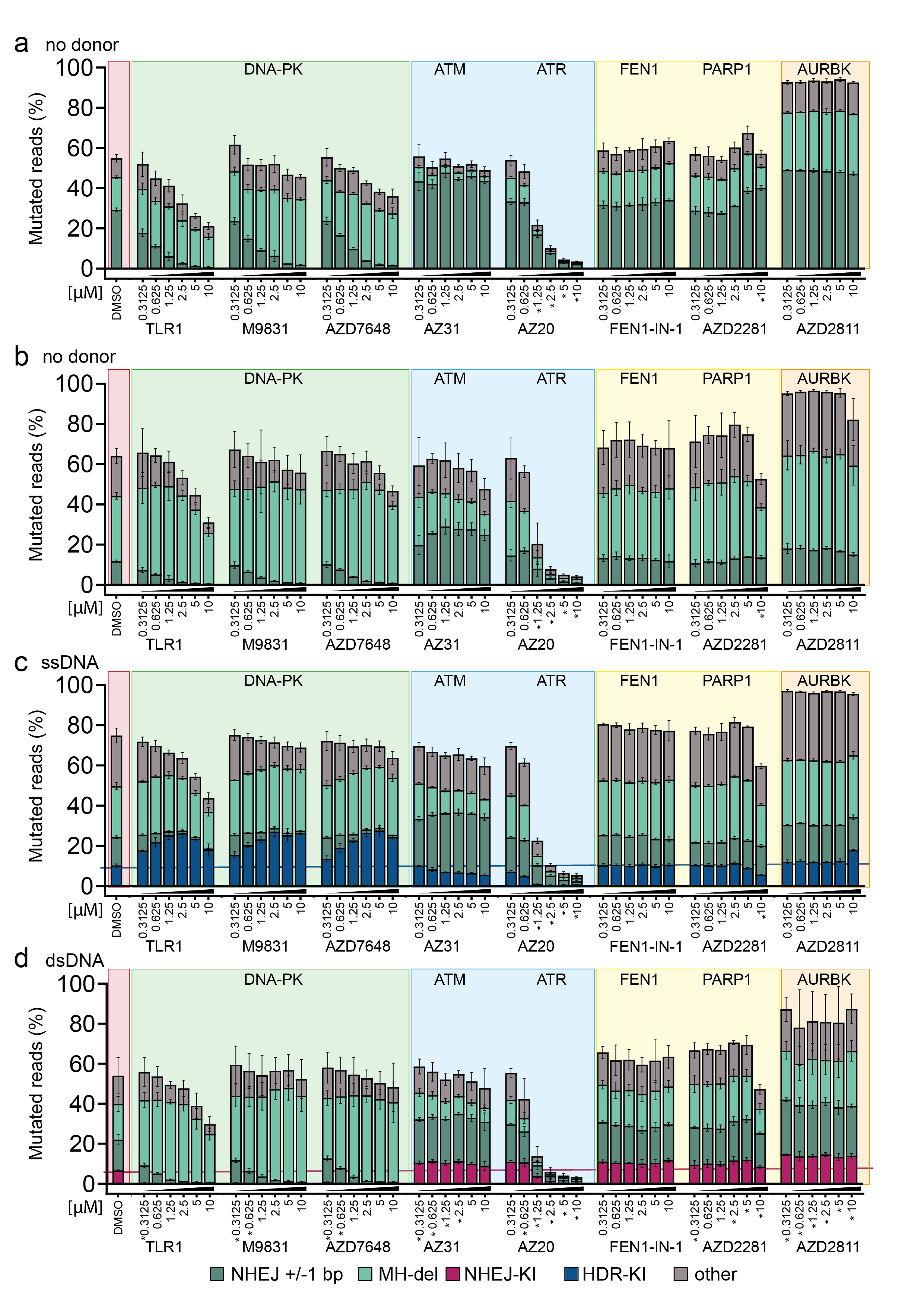


**Supplementary Figure S4 related to Figure 2: Extended analysis of the effect of TLR screen derived inhibitors on DNA repair outcomes at *Sp*Cas9-induced DNA Double-Strand Breaks (DSBs) with Knock-In Sequencing (KI-Seq)** (**a-d**) Fraction of mutated reads by repair pathway in HEK293T cells targeting (**a**) gIns or (**b-d**) gMej *CD34* genomic loci, (**a,b**) without oligo donor, (**c**) with single-stranded DNA (ssDNA) donor or (**d**) double-stranded DNA (dsDNA) donor. KI-Seq was performed on cells treated with different concentrations of DNA repair inhibitors (0.3 – 10 µM) or DMSO control. Horizontal lines represent mean knock-in efficiencies in DMSO treated cells. Compounds and their associated targets are shown. Asterisk indicates treatments affecting cell confluency. Stacked bar graphs depict mean ± standard deviation (n=3, technical replicates). Alterations to the reference DNA sequence are assigned to different DNA DSB repair pathways: +/-1 bp insertions/deletions = non-homologous end joining (NHEJ), deletions with microhomologies (MH-del), *i.e.,* ≥2 bp = alternative end joining (alt-EJ), integration of dsDNA donor without homology arms = NHEJ-KI, integration of a ssDNA donor with homology arms = HDR-KI, all remaining events are summarised in “other”. Source data are provided as a Source Data file.

**Supplementary Figure S5**


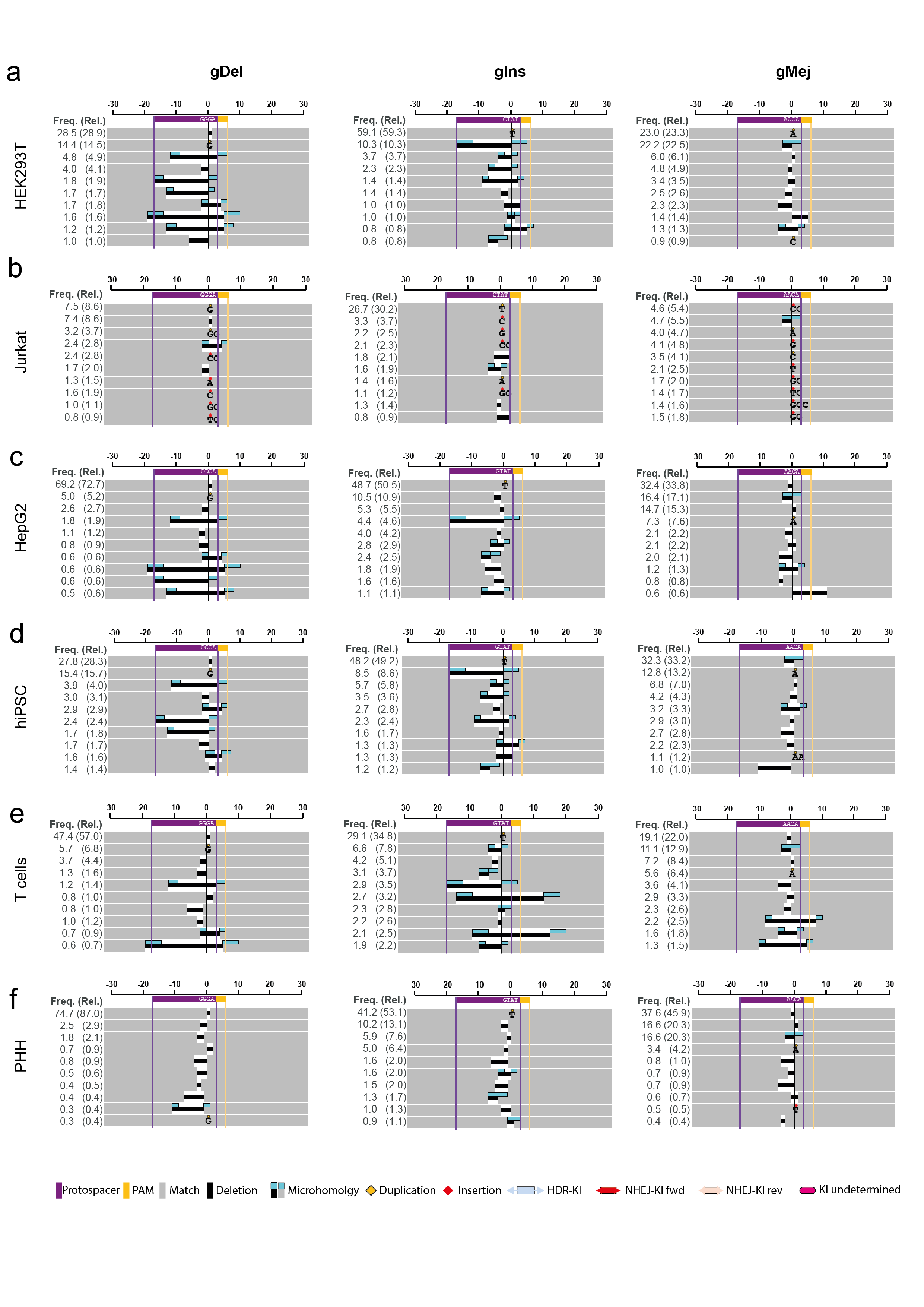


**Supplementary Figure S5 related to Figure 3: Mutation patterns can vary between different cell lines at the same target site.** (**a-f**) Representative InDel profiles +/-30 bp around the *Sp*Cas9 RNP-edited cut site at indicated loci and cell lines. Each variant is annotated with absolute frequencies (Freq.) = fraction of mapped reads and relative frequencies (Rel.) in brackets = fraction of mutated reads. PAM: protospacer adjacent motif; HDR-KI: ssDNA donor with homology arms; NHEJ-KI: dsDNA donor without homology arms; KI undetermined: insertions >10 bp. hiPSC: *Sp*Cas9-inducible human induced pluripotent stem cells; PHH: primary human hepatocytes.

**Supplementary Figure S6**


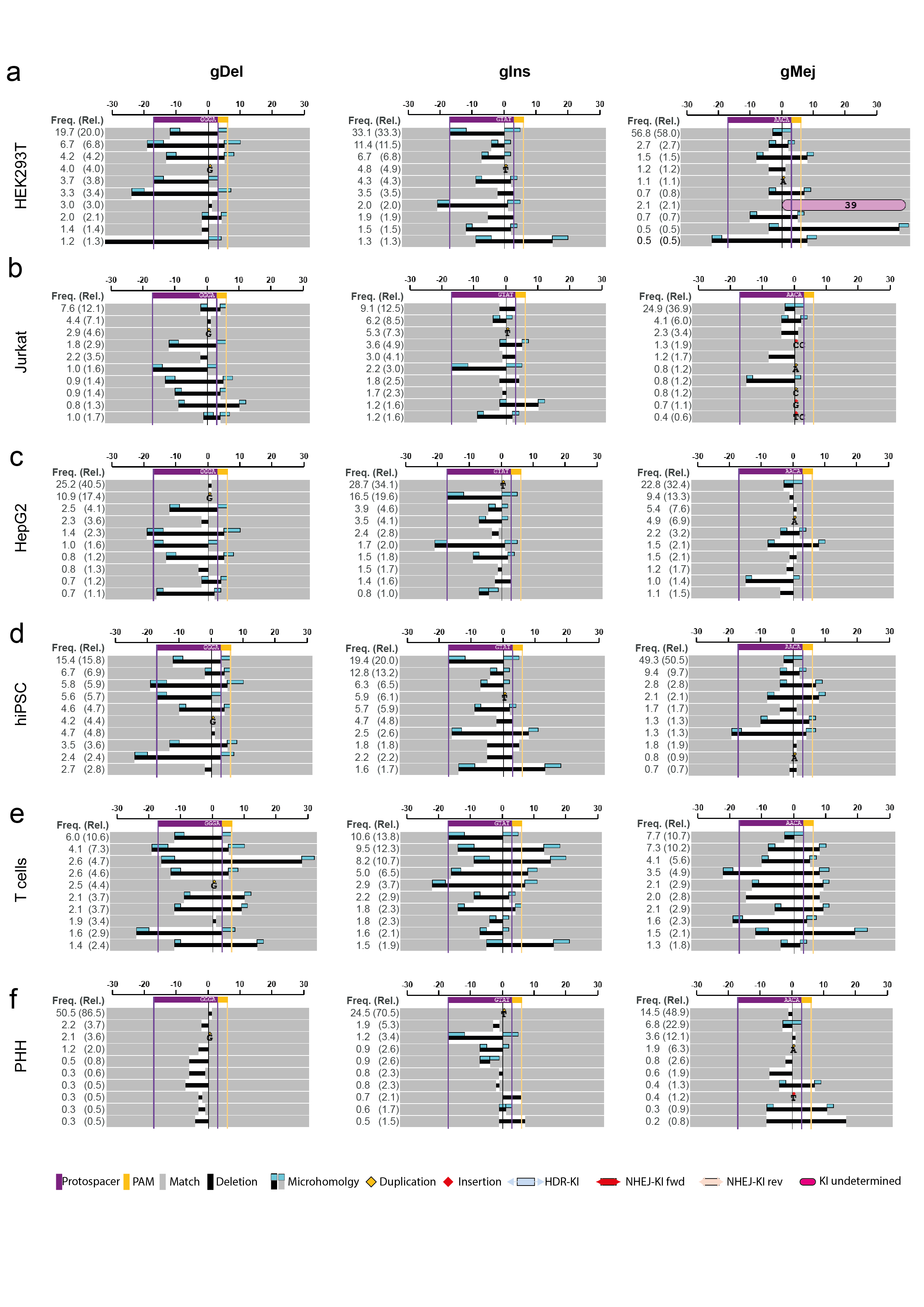


**Supplementary Figure S6 related to Figure 3: DNA-PK inhibition shifts mutation pattern towards longer deletions and increases the frequency of microhomology-associated deletions.** (**a-f**) Representative mutation patterns +/-30 bp around the *Sp*Cas9 RNP-edited cut site at indicated loci and cell types in presence of 1 µM AZD7648. Each variant is annotated with absolute frequencies (Freq.) = fraction of mapped reads and relative frequencies (Rel.) in brackets = fraction of mutated reads. PAM: protospacer adjacent motif; HDR-KI: ssDNA donor with homology arms; NHEJ-KI: dsDNA donor without homology arms; KI undetermined: insertions >10 bp. hiPSC: *Sp*Cas9-inducible human induced pluripotent stem cells; PHH: primary human hepatocytes.

**Supplementary Figure S7**

**
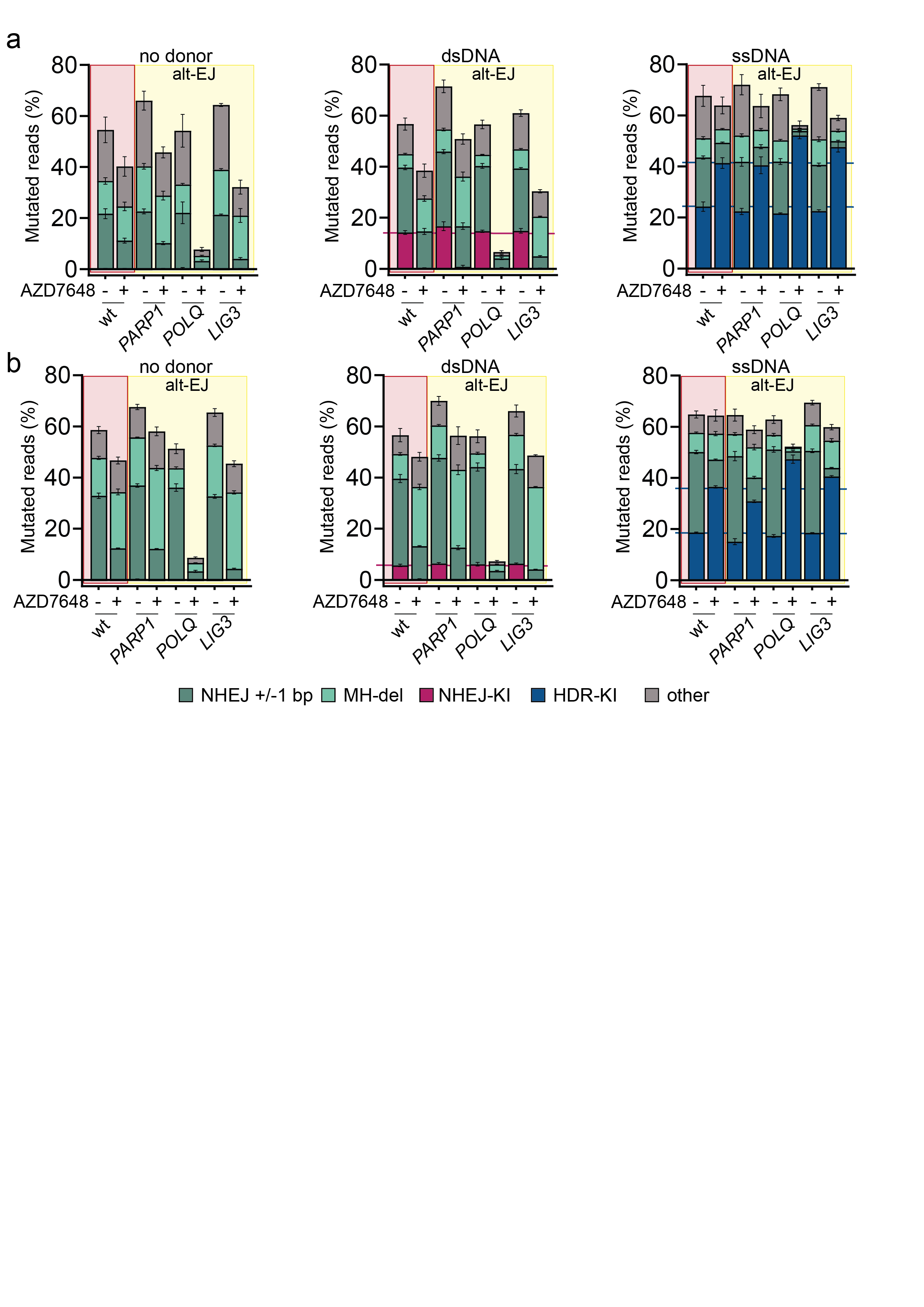
**

**Supplementary Figure S7 related to Figure 4: Simultaneous Polϴ knockout and DNA-PK inhibition increase the frequency and precision of single-stranded DNA (ssDNA)-mediated integration.** (**a,b**) Fraction of mutated reads of different repair events at (a) gDel and (b) gIns target sites with depicted DNA donors for HEK293T wild-type (wt) cells and three knockout cell pools of enzymes involved in alternative end joining (alt-EJ) repair. Cells were treated with 1 µM AZD7648 or DMSO control and transfected with plasmids and ssDNA or dsDNA (double stranded DNA) 3 hours later. Horizontal lines illustrate the mean knock-in efficiency in DMSO or AZD7648 treated wt cells. Bar graphs represent mean values ± standard deviation (n=3, technical replicates). Alterations to the reference DNA sequence are assigned to different DNA DSB repair pathways: +/-1 bp insertions/deletions = non-homologous end joining (NHEJ), deletions with microhomologies (MH-del), *i.e.,* ≥2 bp = alt-EJ, integration of dsDNA donor without homology arms = NHEJ-KI, integration of a ssDNA donor with homology arms = HDR-KI, all remaining events are summarised in “other”. Source data are provided as a Source Data file.

**Supplementary Figure S8**


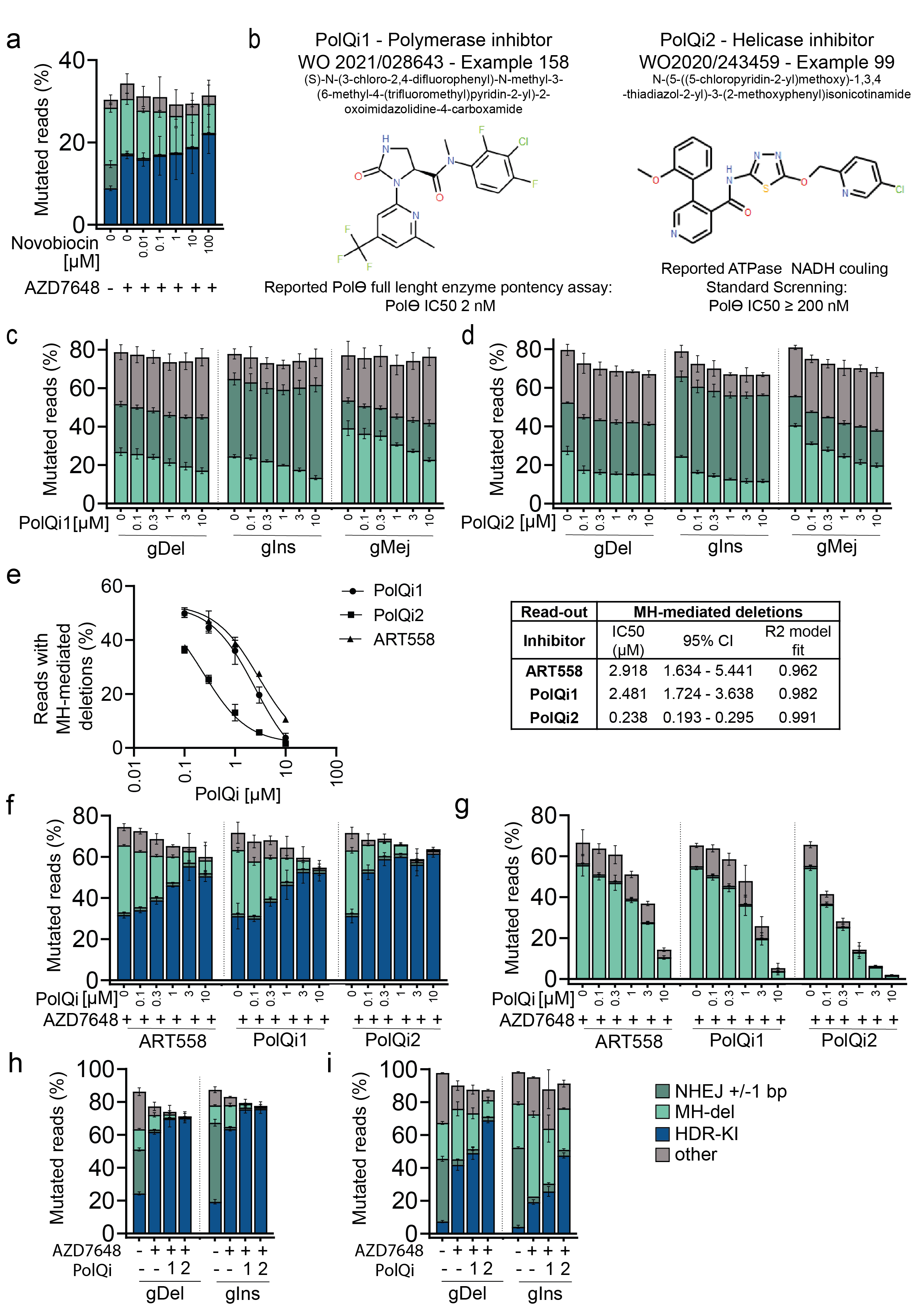


**Supplementary Figure S8 related to Figure 4: Simultaneous inhibition of Polϴ and DNA-PK increases the frequency and precision of single-stranded DNA- (ssDNA) mediated integration.** (**a**) Fraction of mutated reads of different repair events in HEK239T cells, co-treated with 1 µM AZD7648 and increasing concentrations of novobiocin (0.01 – 100 µM), or DMSO control. Cells were treated with compounds and transfected with plasmids and ssDNA 1-3 hours later. Bar graphs show mean values ± standard deviation (n=3, technical replicates). (**b**) Structure of PolQi1 (WO2021/028643, Example 158) and PolQi2 (WO2020/243459, Example 99) and reported biochemical data. (**c,d**) Mutation frequency of different repair events at indicated target sites in HEK239T cells, treated with increasing concentrations of PolQi1 or PolQi2 (0.1 – 10 µM) and DMSO control. Cells were treated with compounds and transfected with plasmids 1-3 hours later. Bar graphs show mean values ± standard deviation (n=3, biological replicates). (**e**) Microhomology- (MH) mediated deletion events in gMej-targeted HEK293T cells treated with several Polϴ inhibitors (0.1 – 10 µM) in the background of 1 µM AZD7648. Dose-response curves show mean values ± standard deviation (n=3, technical replicates). IC50 values were calculated using non-linear regression with a 3-parameter model fit. R2 and 95% confidence interval (CI) are indicated. (**f,g**) Dose-dependent effect on the distribution of DNA repair events in gMej-targeted HEK293T cells treated with several Polϴ inhibitors (0.1 – 10 µM) in the presence of 1 µM AZD7648 1-3 hours before transfection (f) with and (g) without ssDNA. Bar graphs illustrate mean fraction of mutated reads ± SD (n=3, technical replicates). (**h**) Frequencies of different repair events at gDel and gIns target sites in HEK293T cells co-transfected with ssDNA, *Sp*Cas9 and sgRNA plasmids. Cells were treated 1-3 hours before transfections with DMSO, 1 µM DNA-PK inhibitor AZD7648, and 1 µM AZD7648 in combination with 3 µM Polϴ inhibitor (PolQi) 1 or 2. Bar graphs represent mean percentage of mutated reads ± standard deviation (n=3, biological replicates). (**i**) Bar graphs depict the mean percentage of mutated reads of different repair events ± standard deviation (n=3, technical replicates) in *Sp*Cas9-inducible human induced pluripotent stem cells (hiPSC) transfected with gDel and gIns in the presence of DMSO, 1 µM AZD7648, and 1 µM AZD7648 in combination with 3 µM PolQi1 or PolQi2. Alterations to the reference DNA sequence are assigned to different DNA DSB repair pathways: +/-1 bp insertions/deletions = non-homologous end joining (NHEJ), deletions with microhomologies (MH-del), *i.e.,* ≥2 bp = alternative end joining (alt-EJ), integration of a ssDNA donor with homology arms = HDR-KI, all remaining events are summarised in “other”. Source data are provided as a Source Data file.

**Supplementary Figure S9**


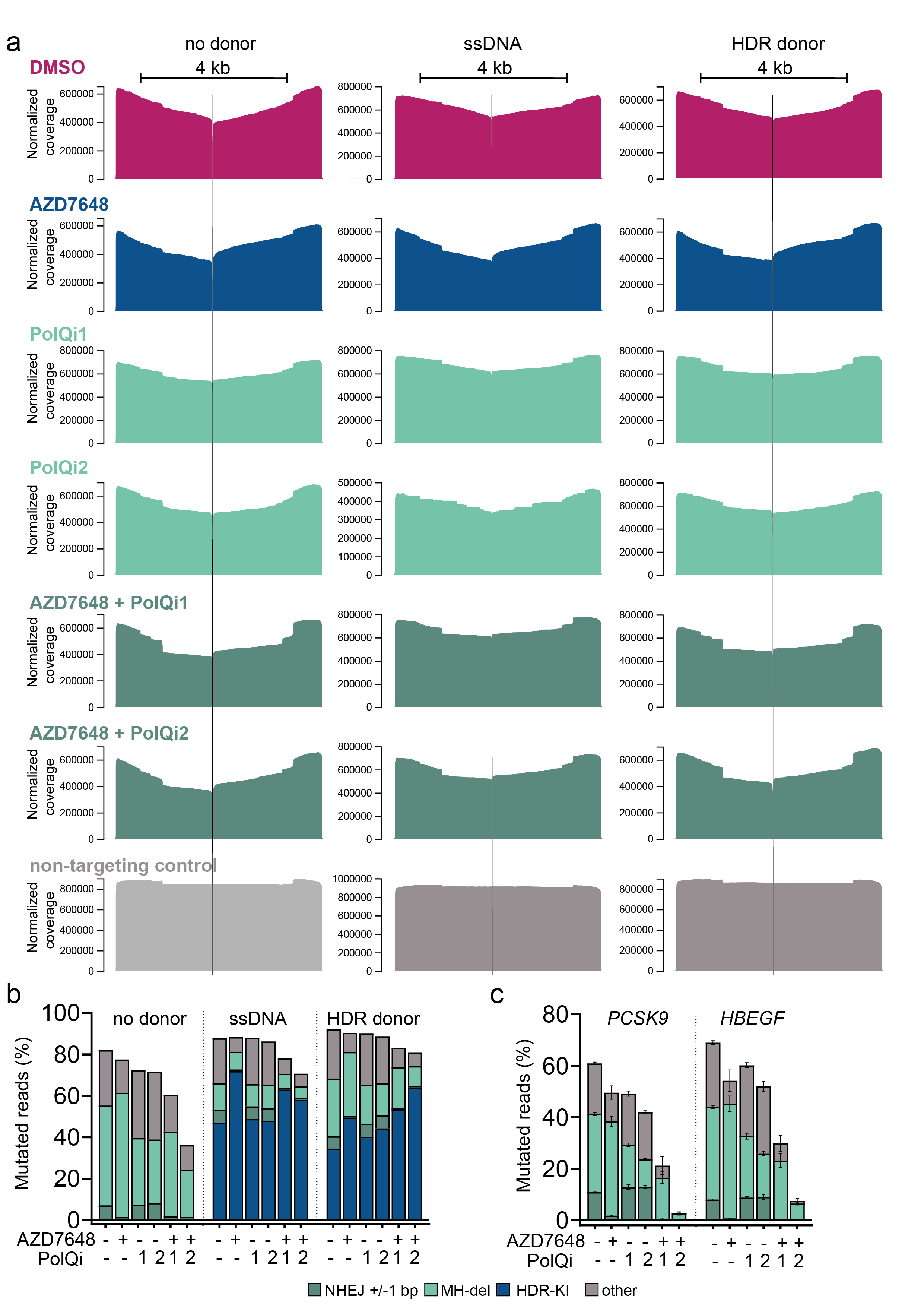


**Supplementary Figure S9 related to Figure 5: 2iHDR retains large deletions frequencies and decreases translocations.** (**a**) Coverage of long-range sequencing reads aligned to the edited *HBEGF* locus normalized to counts per million reads. Treatments with DMSO or small molecules (1 µM AZD7648, 3 µM PolQi1 and 3 µM PolQi2) and knock-in donors were used as depicted. (**b**) Fraction of mutated reads of different repair events for samples used in large deletion experiments targeting *HBEGF* with DNA donors depicted, analysed with Knock-In Sequencing **(**KI-Seq). Cells were treated with indicated inhibitors (1 µM AZD7648, 3 µM PolQi1 and 3 µM PolQi2) or DMSO control (n=1). ssDNA: single-stranded DNA donor with homology arms; HDR donor: plasmid with long homology arms. (**c**) Bar graphs show mean fraction of mutated reads of different repair events for samples used in translocation experiments ± standard deviation (n=3, technical replicates). KI-Seq was performed for the indicated loci in presence of DMSO control or small molecules (1 µM AZD7648, 3 µM PolQi1, or 3 µM PolQi2). Alterations to the reference DNA sequence are assigned to different DNA DSB repair pathways: +/-1 bp insertions/deletions = non-homologous end joining (NHEJ), deletions with microhomologies (MH-del), *i.e.,* ≥2 bp = alternative end joining (alt-EJ), integration of a ssDNA donor with homology arms = HDR-KI, all remaining events are summarised in “other”. Source data are provided as a Source Data file.

**Supplementary Figure S10**


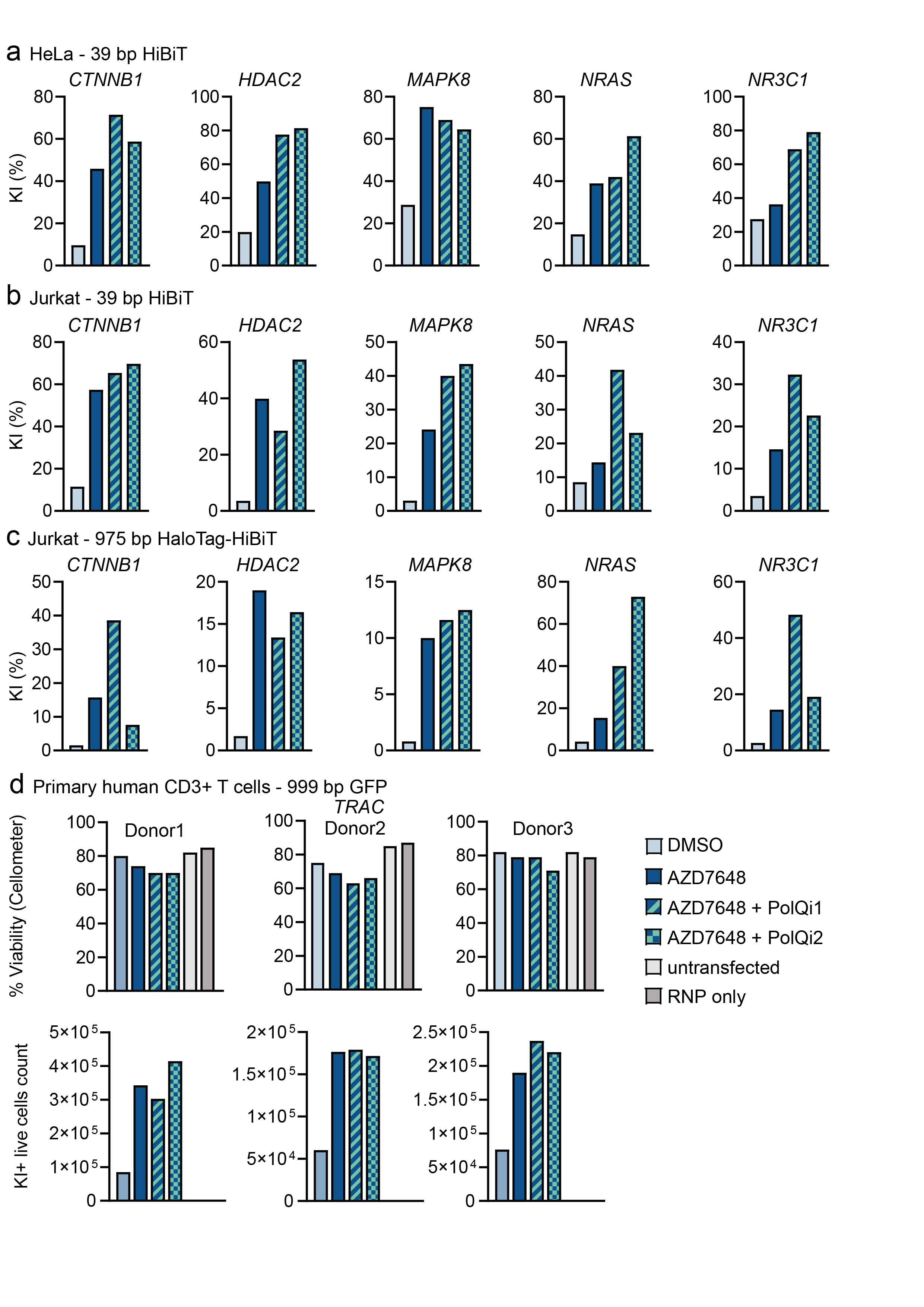


**Supplementary Figure S10 related to Figure 6: 2iHDR increases integration of different DNA donor templates with various integration-to-cut-site distances in diverse cell types. Validation of integration with droplet digital PCR (ddPCR) and cell viability measurement.** (**a-c**) Quantification of HiBiT and HaloTag-HiBiT integration assessed with ddPCR at indicated genomic loci and cell lines. Treatment with 1 µM AZD7648 is compared to 2iHDR treatment (1 µM AZD7648 and 3 µM PolQi1 or PolQi2) or DMSO control. Bar graphs represent integration efficiency in percent (n=1). (**d**) Bar graphs show the percentage of viable CD3+ T cells assessed with trypan blue (upper panel) and corresponding live cell counts of knock-in containing cells (KI+) (lower panel). Cells were treated with DMSO, 1 µM AZD7648 or a combination of 1 µM AZD7648 and 3 µM PolQi1 or PolQi2 and compared to untreated or ribonucleoprotein (RNP) complex only treated controls as indicated.

**Supplementary Figure S11.**


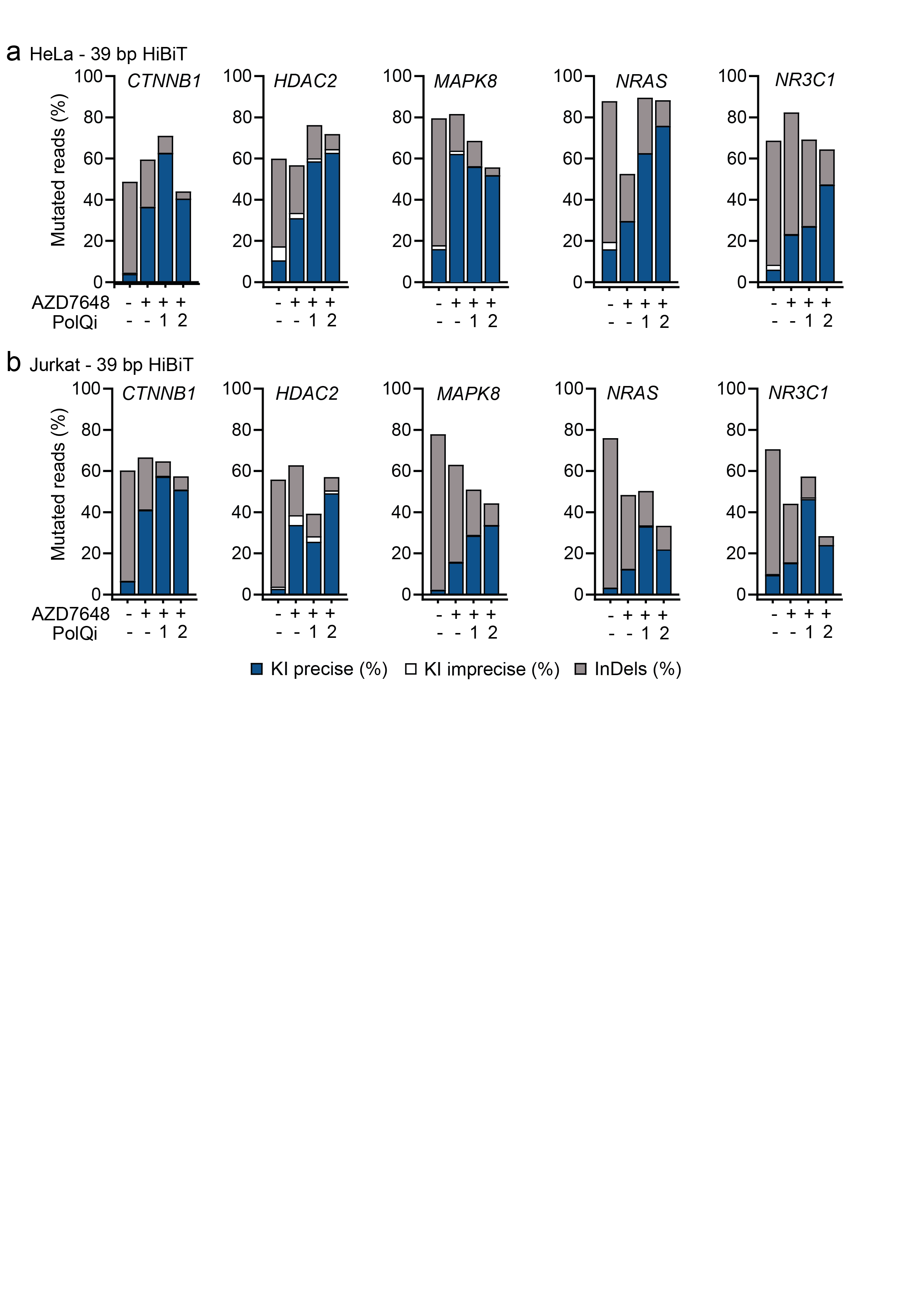


**Supplementary Figure S11 related to Figure 6: 2iHDR increases integration of different DNA donor templates with various integration-to-cut-site distances in diverse cell types.** Graphs show results of deep-targeted amplicon sequencing. (**a,b**) Representative fraction of mutated reads of different repair events in indicated cell types at various target sites evaluated with CRISPResso2 applying a quantification window of one (n=1). Knock-in precise = perfect HiBiT insertion, knock-in imprecise = HiBiT integration with additional mutations, InDels = all additional mutations independent from HiBiT integration. Experiments compare treatments with DMSO control, 1 µM AZD7648 and 2iHDR (1 µM AZD7648 and 3 µM PolQi1 or PolQi2).

**Supplementary Figure S12**

**
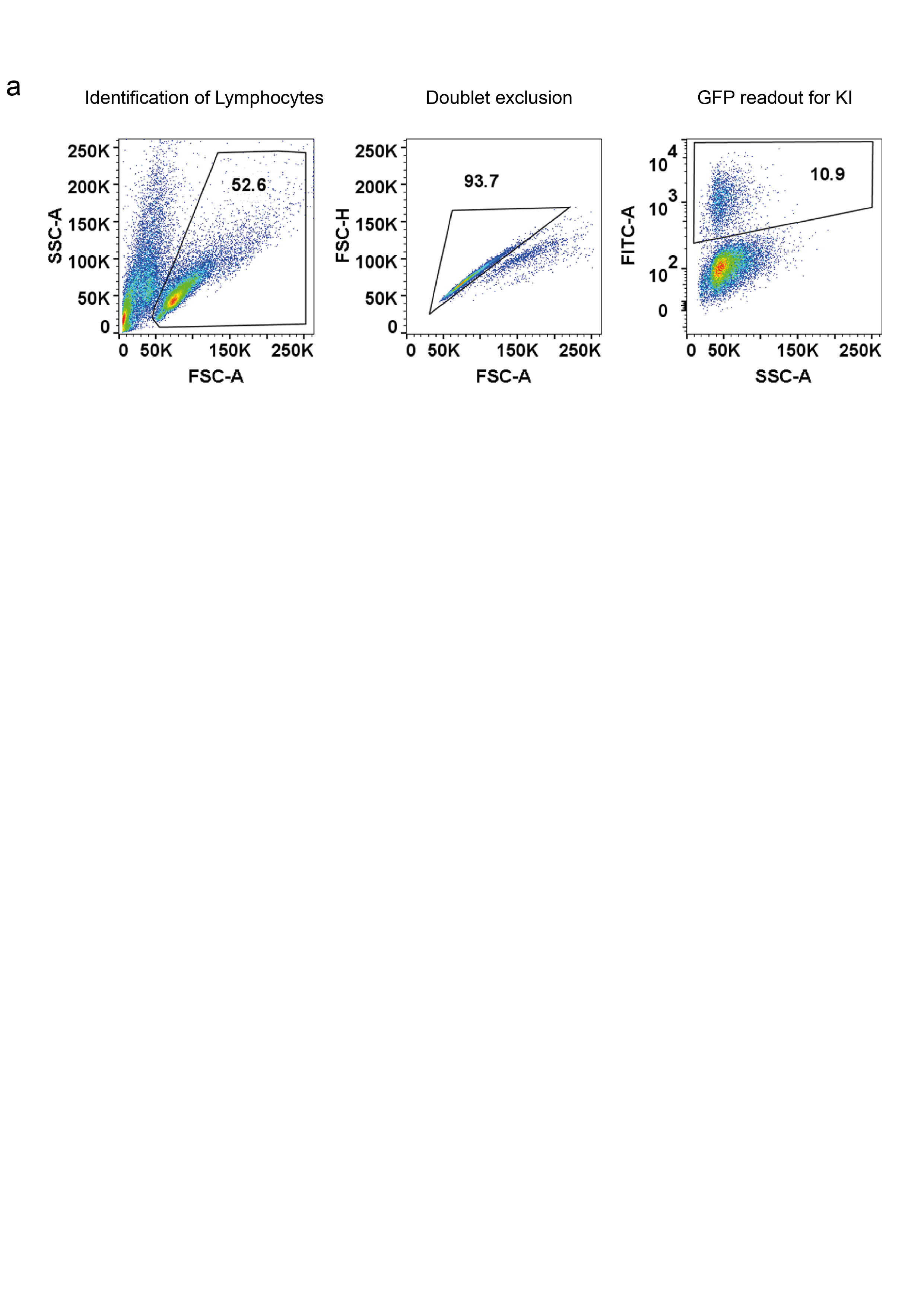
**

**Supplementary Figure S12 related to Figure 6: 2iHDR increases DNA template integration in primary human CD3+ T cells.** (**a**) Representative flow cytometry plots to illustrate the gating strategy to measure knock-in (KI) efficiency in primary human CD3+ T cells. Lymphocytes were selected based on forward scatter (FSC) and side scatter (SSC), and subsequently gated on singlets with FSC height (FSC-H) and FSC-area (FSC-A) (doublet exclusion), followed by GFP quantification to measure KI efficiency.

**Supplementary Note 1.** Plasmids used in this study.

**CMV-SpCas9-2AGFP**

CMV promoter
SpCas9
T2A
GFP
Bovine Growth Hormone Polyadenylation (BGH-PolyA) signal
Kanamycin resistance
Backbone

CCCGTAGAAAAGATCAAAGGATCTTCTTGAGATCCTTTTTTTCTGCGCGTAATCTGCTGCTTGCAAACAAAAAAACCACCGCTACCAGCGGTGGTTTGTTTGCCGGATCAAGAGCTACCAACTCTTTTTCCGAAGGTAACTGGCTTCAGCAGAGCGCAGATACCAAATACTGTCCTTCTAGTGTAGCCGTAGTTAGGCCACCACTTCAAGAACTCTGTAGCACCGCCTACATACCTCGCTCTGCTAATCCTGTTACCAGTGGCTGCTGCCAGTGGCGATAAGTCGTGTCTTACCGGGTTGGACTCAAGACGATAGTTACCGGATAAGGCGCAGCGGTCGGGCTGAACGGGGGGTTCGTGCACACAGCCCAGCTTGGAGCGAACGACCTACACCGAACTGAGATACCTACAGCGTGAGCTATGAGAAAGCGCCACGCTTCCCGAAGGGAGAAAGGCGGACAGGTATCCGGTAAGCGGCAGGGTCGGAACAGGAGAGCGCACGAGGGAGCTTCCAGGGGGAAACGCCTGGTATCTTTATAGTCCTGTCGGGTTTCGCCACCTCTGACTTGAGCGTCGATTTTTGTGATGCTCGTCAGGGGGGCGGAGCCTATGGAAAAACGCCAGCAACGCGGCCTTTTTACGGTTCCTGGGCTTTTGCTGGCCTTTTGCTCACATGTTCTTGCTGCTTCGCGATGTACGGGCCAGATATA**CGCGTTGACATTGATTATTGACTAGTTATTAATAGTAATCAATTACGGGGTCATTAGTTCATAGCCCATATATGGAGTTCCGCGTTACATAACTTACGGTAAATGGCCCGCCTGGCTGACCGCCCAACGACCCCCGCCCATTGACGTCAATAATGACGTATGTTCCCATAGTAACGCCAATAGGGACTTTCCATTGACGTCAATGGGTGGACTATTTACGGTAAACTGCCCACTTGGCAGTACATCAAGTGTATCATATGCCAAGTACGCCCCCTATTGACGTCAATGACGGTAAATGGCCCGCCTGGCATTATGCCCAGTACATGACCTTATGGGACTTTCCTACTTGGCAGTACATCTACGTATTAGTCATCGCTATTACCATGGTGATGCGGTTTTGGCAGTACATCAATGGGCGTGGATAGCGGTTTGACTCACGGGGATTTCCAAGTCTCCACCCCATTGACGTCAATGGGAGTTTGTTTTGGCACCAAAATCAACGGGACTTTCCAAAATGTCGTAACAACTCCGCCCCATTGACGCAAATGGGCGGTAGGCGTGTACGGTGGGAGGTCTATATAAGCAGAGCTC**TCTGGCTAACTAGAGAACCCACTGCTTACTGGCTTATCGAAATTAATACGACTCACTATAGGGAGACCCAAGCTGGCTAGCGTTTAAACTTAAGCTGATCCACTAGTCCAGTGTGGTGGAATTCGCC**ATGGCTCCTAAGAAAAAGCGGAAGGTGGACAAGAAATACTCAATCGGGCTGGACATCGGAACTAACTCAGTGGGGTGGGCAGTCATTACTGACGAGTACAAAGTGCCAAGCAAGAAATTTAAGGTCCTGGGCAACACCGATAGGCACTCCATCAAGAAAAATCTGATTGGGGCCCTGCTGTTCGACTCTGGAGAGACAGCTGAAGCAACTAGACTGAAAAGGACTGCTAGAAGGCGCTATACCCGGCGAAAGAATCGCATCTGCTACCTGCAGGAGATTTTCTCTAACGAAATGGCCAAGGTGGACGATAGTTTCTTTCATCGGCTGGAGGAATCATTCCTGGTCGAGGAAGATAAGAAACACGAGAGACATCCTATCTTTGGAAACATTGTGGACGAGGTCGCTTATCACGAAAAATACCCCACCATCTATCATCTGCGCAAGAAACTGGTGGACTCTACAGATAAAGCAGACCTGCGGCTGATCTATCTGGCCCTGGCTCACATGATTAAGTTCAGAGGCCATTTTCTGATCGAGGGAGATCTGAACCCAGACAATAGCGATGTGGACAAGCTGTTCATCCAGCTGGTCCAGACATACAATCAGCTGTTTGAGGAAAACCCTATTAATGCATCTGGCGTGGACGCAAAAGCCATCCTGAGTGCCAGGCTGTCTAAGAGTAGAAGGCTGGAGAACCTGATCGCTCAGCTGCCAGGCGAAAAGAAAAACGGCCTGTTTGGAAATCTGATTGCACTGTCACTGGGACTGACACCTAACTTCAAGAGCAATTTTGATCTGGCCGAGGACGCTAAACTGCAGCTGAGCAAGGACACTTATGACGATGACCTGGATAACCTGCTGGCTCAGATCGGAGATCAGTACGCAGACCTGTTCCTGGCCGCTAAGAATCTGTCTGACGCTATCCTGCTGAGTGATATTCTGCGGGTGAACACCGAGATTACAAAAGCCCCTCTGTCAGCTAGCATGATCAAGAGATATGACGAGCACCATCAGGATCTGACCCTGCTGAAGGCACTGGTGCGCCAGCAGCTGCCCGAGAAGTACAAGGAAATCTTCTTTGATCAGAGTAAGAACGGGTACGCCGGTTATATTGACGGCGGAGCTTCACAGGAGGAATTCTACAAGTTTATCAAACCTATTCTGGAGAAGATGGACGGCACCGAGGAACTGCTGGTGAAACTGAATCGCGAGGACCTGCTGCGCAAGCAGCGGACATTTGATAACGGCTCCATCCCCCACCAGATTCATCTGGGAGAGCTGCACGCAATCCTGCGACGACAGGAAGACTTCTACCCATTTCTGAAGGATAACCGCGAGAAGATCGAAAAAATTCTGACCTTCCGGATCCCTTACTATGTGGGGCCCCTGGCAAGGGGTAATTCCCGCTTTGCCTGGATGACACGGAAATCTGAGGAAACAATCACTCCTTGGAACTTCGAGGAAGTGGTCGATAAGGGAGCTTCCGCACAGTCTTTCATCGAGAGAATGACAAACTTCGACAAAAACCTGCCAAATGAGAAAGTGCTGCCTAAGCACAGTCTGCTGTACGAGTATTTCACAGTCTATAACGAACTGACTAAGGTGAAATACGTCACCGAGGGGATGAGGAAGCCCGCCTTCCTGAGCGGTGAACAGAAGAAAGCTATCGTGGACCTGCTGTTTAAAACCAATCGCAAGGTGACAGTCAAGCAGCTGAAGGAGGACTACTTCAAGAAAATTGAATGTTTCGATTCTGTGGAGATCAGTGGCGTCGAAGACAGATTTAACGCTTCTCTGGGAACCTACCACGATCTGCTGAAGATCATTAAGGATAAAGACTTCCTGGACAACGAGGAAAATGAGGATATCCTGGAAGACATTGTGCTGACCCTGACACTGTTTGAGGATCGCGAAATGATCGAGGAACGGCTGAAAACTTATGCCCATCTGTTCGATGACAAGGTGATGAAACAGCTGAAGCGAAGAAGGTACACCGGCTGGGGACGACTGAGCAGAAAGCTGATCAACGGCATTCGGGACAAACAGAGTGGAAAGACTATCCTGGACTTTCTGAAATCAGATGGCTTCGCTAACAGAAATTTTATGCAGCTGATTCACGATGACAGCCTGACCTTCAAAGAGGATATCCAGAAGGCACAGGTGTCCGGGCAGGGTGACTCTCTGCACGAGCATATCGCAAACCTGGCCGGGTCCCCCGCCATCAAGAAAGGTATTCTGCAGACCGTGAAGGTGGTCGATGAGCTGGTGAAAGTCATGGGCAGGCATAAGCCAGAAAACATCGTGATTGAGATGGCCCGCGAAAATCAGACCACACAGAAAGGACAGAAGAACAGCCGCGAGCGGATGAAAAGGATCGAGGAAGGCATTAAGGAACTGGGATCCCAGATCCTGAAAGAGCACCCTGTGGAAAACACTCAGCTGCAGAATGAGAAGCTGTATCTGTACTATCTGCAGAATGGGCGGGATATGTACGTGGACCAGGAGCTGGATATTAACCGACTGTCTGATTACGACGTGGATCATATCGTCCCACAGTCATTCCTGAAAGATGACAGCATTGACAATAAGGTGCTGACCCGGAGTGACAAAAACCGAGGAAAGAGTGATAATGTCCCTTCAGAGGAAGTGGTCAAGAAAATGAAGAACTACTGGAGACAGCTGCTGAATGCCAAACTGATCACACAGCGAAAGTTTGATAACCTGACTAAAGCTGAGAGAGGGGGTCTGTCAGAACTGGACAAAGCAGGCTTCATCAAGCGACAGCTGGTGGAGACCAGACAGATCACAAAGCACGTCGCTCAGATTCTGGATAGCAGGATGAACACAAAGTACGATGAGAATGACAAACTGATCCGCGAAGTGAAGGTCATTACTCTGAAGTCAAAACTTGTGAGCGACTTCAGAAAGGATTTCCAGTTCTACAAAGTCAGGGAGATCAACAATTATCACCATGCTCATGACGCATACCTGAACGCAGTGGTCGGGACCGCCCTGATTAAGAAATACCCCAAACTGGAGAGCGAATTCGTGTACGGTGACTATAAGGTGTACGATGTCAGAAAAATGATCGCCAAGAGTGAGCAGGAAATTGGAAAAGCCACCGCTAAGTATTTCTTTTACTCAAACATCATGAATTTCTTTAAGACTGAGATCACCCTGGCAAATGGGGAAATCCGAAAGAGACCACTGATTGAGACTAACGGCGAGACCGGAGAAATCGTGTGGGACAAGGGTAGGGATTTTGCCACAGTGCGCAAGGTCCTGTCCATGCCTCAAGTGAATATTGTCAAGAAAACAGAGGTGCAGACTGGCGGATTCAGTAAGGAATCAATTCTGCCCAAACGGAACTCTGATAAGCTGATCGCCCGAAAGAAAGACTGGGATCCCAAGAAATATGGGGGTTTCGACTCCCCAACAGTGGCTTACTCTGTCCTGGTGGTCGCAAAGGTGGAGAAGGGGAAAAGCAAGAAACTGAAATCCGTCAAGGAGCTGCTGGGTATCACTATTATGGAGAGGAGCTCCTTCGAGAAGAACCCCATCGATTTTCTGGAGGCTAAAGGCTATAAGGAAGTGAAGAAAGACCTGATCATTAAACTGCCAAAGTACAGCCTGTTTGAGCTGGAAAACGGAAGGAAGCGAATGCTGGCATCCGCAGGAGAGCTGCAGAAGGGTAATGAACTGGCCCTGCCTTCTAAGTACGTGAACTTCCTGTATCTGGCTAGCCACTACGAGAAGCTGAAAGGCTCCCCCGAGGATAACGAACAGAAACAGCTGTTTGTGGAGCAGCACAAGCATTATCTGGACGAGATCATTGAACAGATTAGCGAGTTCTCCAAAAGAGTGATCCTGGCTGACGCAAATCTGGATAAGGTCCTGAGCGCATACAACAAACACAGAGATAAGCCAATCAGGGAGCAGGCCGAAAATATCATTCATCTGTTCACTCTGACCAACCTGGGAGCCCCTGCAGCCTTCAAGTATTTTGACACTACCATCGATCGGAAACGATACACATCCACTAAGGAGGTGCTGGACGCTACCCTGATTCACCAGAGCATTACCGGCCTGTATGAAACAAGGATTGACCTGTCTCAGCTGGGGGGCGACCTCGAGGATGGGGACGAGGGCAGAGGAAGTCTGCTAACATGCGGTGACGTCGAGGAGAATCCTGGCCCAGCACCGGGATCCATGGTGAGCAAGGGCGAGGAGCTGTTCACCGGGGTGGTGCCCATCCTGGTCGAGCTGGACGGCGACGTAAACGGCCACAAGTTCAGCGTGTCCGGCGAGGGCGAGGGCGATGCCACCTACGGCAAGCTGACCCTGAAGTTCATCTGCACCACCGGCAAGCTGCCCGTGCCCTGGCCCACCCTCGTGACCACCTTCACCTACGGCGTGCAGTGCTTCGCCCGCTACCCCGACCACATGAAGCAGCACGACTTCTTCAAGTCCGCCATGCCCGAAGGCTACGTCCAGGAGCGCACCATCTTCTTCAAGGACGACGGCAACTACAAGACCCGCGCCGAGGTGAAGTTCGAGGGCGACACCCTGGTGAACCGCATCGAGCTGAAGGGCATCGACTTCAAGGAGGACGGCAACATCCTGGGGCACAAGCTGGAGTACAACTACAACAGCCACAAGGTCTATATCACCGCCGACAAGCAGAAGAACGGCATCAAGGTGAACTTCAAGACCCGCCACAACATCGAGGACGGCAGCGTGCAGCTCGCCGACCACTACCAGCAGAACACCCCCATCGGCGACGGCCCCGTGCTGCTGCCCGACAACCACTACCTGAGCACCCAGTCCGCCCTGAGCAAAGACCCCAACGAGAAGCGCGATCACATGGTCCTGCTGGAGTTCGTGACCGCCGCCGGGATCACTCTCGGCATGGACGAGCTGTACAAGTGATA**ATCTAGAGGGCCCGTTTAAACCCGCTGATCAGCCTCGA**CTGTGCCTTCTAGTTGCCAGCCATCTGTTGTTTGCCCCTCCCCCGTGCCTTCCTTGACCCTGGAAGGTGCCACTCCCACTGTCCTTTCCTAATAAAATGAGGAAATTGCATCGCATTGTCTGAGTAGGTGTCATTCTATTCTGGGGGGTGGGGTGGGGCAGGACAGCAAGGGGGAGGATTGGGAAGACAATAGCAGGCATGCTGGGGATGCGGTGGGCTCTATGG**CTTCTACTGGGCGGTTTTATGGACAGCAAGCGAACCGGAATTGCCAGCTGGGGCGCCCTCTGGTAAGGTTGGGAAGCCCTGCAAAGTAAACTGGATGGCTTTCTCGCCGCCAAGGATCTGATGGCGCAGGGGATCAAGCTCTGATCAAGAGACAGGATGAGGATCGTTTCGC**ATGATTGAACAAGATGGATTGCACGCAGGTTCTCCGGCCGCTTGGGTGGAGAGGCTATTCGGCTATGACTGGGCACAACAGACAATCGGCTGCTCTGATGCCGCCGTGTTCCGGCTGTCAGCGCAGGGGCGCCCGGTTCTTTTTGTCAAGACCGACCTGTCCGGTGCCCTGAATGAACTGCAAGACGAGGCAGCGCGGCTATCGTGGCTGGCCACGACGGGCGTTCCTTGCGCAGCTGTGCTCGACGTTGTCACTGAAGCGGGAAGGGACTGGCTGCTATTGGGCGAAGTGCCGGGGCAGGATCTCCTGTCATCTCACCTTGCTCCTGCCGAGAAAGTATCCATCATGGCTGATGCAATGCGGCGGCTGCATACGCTTGATCCGGCTACCTGCCCATTCGACCACCAAGCGAAACATCGCATCGAGCGAGCACGTACTCGGATGGAAGCCGGTCTTGTCGATCAGGATGATCTGGACGAAGAGCATCAGGGGCTCGCGCCAGCCGAACTGTTCGCCAGGCTCAAGGCGAGCATGCCCGACGGCGAGGATCTCGTCGTGACCCATGGCGATGCCTGCTTGCCGAATATCATGGTGGAAAATGGCCGCTTTTCTGGATTCATCGACTGTGGCCGGCTGGGTGTGGCGGACCGCTATCAGGACATAGCGTTGGCTACCCGTGATATTGCTGAAGAGCTTGGCGGCGAATGGGCTGACCGCTTCCTCGTGCTTTACGGTATCGCCGCTCCCGATTCGCAGCGCATCGCCTTCTATCGCCTTCTTGACGAGTTCTTCTGA**ATTATTAACGCTTACAATTTCCTGATGCGGTATTTTCTCCTTACGCATCTGTGCGGTATTTCACACCGCATACAGGTGGCACTTTTCGGGGAAATGTGCGCGGAACCCCTATTTGTTTATTTTTCTAAATACATTCAAATATGTATCCGCTCATGAGACAATAACCCTGATAAATGCTTCAATAATAGCACGTGCTAAAACTTCATTTTTAATTTAAAAGGATCTAGGTGAAGATCCTTTTTGATAATCTCATGACCAAAATCCCTTAACGTGAGTTTTCGTTCCACTGAGCGTCAGAC

**pMLU sgRNA backbone**

U6 Promoter
Scaffold
Terminator
Stuffer
Kanamycin resistance
Backbone

**TGTACAAAAAAGCAGGCTTTAAAGGAACCAATTCAGTCGACTGGATCCGGTACCAAGGTCGGGCAGGAAGAGGGCCTATTTCCCATGATTCCTTCATATTTGCATATACGATACAAGGCTGTTAGAGAGATAATTAGAATTAATTTGACTGTAAACACAAAGATATTAGTACAAAATACGTGACGTAGAAAGTAATAATTTCTTGGGTAGTTTGCAGTTTTAAAATTATGTTTTAAAATGGACTATCATATGCTTACCGTAACTTGAAAGTATTTCGATTTCTTGGCTTTATATATCTTGTGGAAAGGACGAAACACCGTATCGCAGGTGATTCTCTAGACATCATTAATTCCTAATTTTTGTTGACACTCTATCATTGATAGAGTTATTTTACCACTCCCTATCAGTGATAGAGAAAAGTGAAATGGCAACTGTTAACCAGCTGGTGCGCAAACCGCGTGCGCGTAAGGTTGCAAAAAGCAACGTTCCTGCTCTGGAAGCTTGCCCGCAGAAGCGTGGTGTATGTACTCGCGTATATACCACCACCCCTAAAAAACCAAACTCTGCACTGCGTAAAGTTTGTCGTGTGCGTCTGACTAACGGCTTCGAAGTTACCTCTTACATCGGTGGCGAAGGCCACAACCTGCAAGAACACTCCGTAATTCTGATCCGTGGTGGCCGTGTAAAAGACCTCCCAGGTGTGCGTTACCACACTGTTCGCGGTGCACTGGACTGTTCCGGTGTTAAAGACCGTAAACAAGCGCGTTCTAAGTACGGTGTGAAGCGTCCAAAAGCTTAAAGGAGGACAATCATGGCCAAGCCTTTGTCTCAAGAAGAATCCACCCTCATTGAAAGAGCAACGGCTACAATCAACAGCATCCCCATCTCTGAAGACTACAGCGTCGCCAGCGCAGCTCTCTCTAGCGACGGCCGCATCTTCACTGGTGTCAATGTATATCATTTTACTGGGGGACCTTGTGCAGAACTCGTGGTGCTGGGCACTGCTGCTGCTGCGGCAGCTGGCAACCTGACTTGTATCGTCGCGATCGGAAATGAGAACAGGGGCATCTTGAGCCCCTGCGGACGGTGTCGACAGGTGCTTCTCGATCTGCATCCTGGGATCAAAGCGATAGTGAAGGACAGTGATGGACAGCCGACGGCAGTTGGGATTCGTGAATTGCTGCCCTCTGGTTATGTGTGGGAGGGCTAACACCTGCATCCGTTTTAGAGCTAGAAATAGCAAGTTAAAATAAGGCTAGTCCGTTATCAACTTGAAAAAGTGGCACCGAGTCGGTGCTTTTTTT**CTAGACCCAGCTTTCTTGTACAAAGTTGGCATTAACGCGTTGACATTGATTATTGACTAGCCACTACGAGAAGCTGAAAGGCTCCCCCGAGGATAACGAACAGAAACAGCTGTTTGTGGAGCAGCACAAGCATTATCTGGACGAGATCATTGAACAGATTAGCGAGTTCTCCAAAAGAGTGATCCTGGCTGACGCAAATCTGGATAAGGTCCTGAGCGCATACAACAAACACAGAGATAAGCCAATCAGGGAGCAGGCCGAAAATATCATTCATCTGTTCACTCTGACCAACCTGGGAGCCCCTGCAGCCTTCAAGTATTTTGACACTACCATCGATCGGAAACGATACACATCCACTAAGGAGGTGCTGGACGCTACCCTGATTCACCAGAGCATTACCGGCCTGTATGAAACAAGGATTGACCTGTCTCAGCTGGGCGGCGACAGCAGGGCTGACCCCAAGAAGAAGAGGAAGGTGTGATAACTCGAGTCTAGAGGGCCCGTTTAAACCCGCTGATCAGCCTCGACTGTGCCTTCTAGTTGCCAGCCATCTGTTGTTTGCCCCTCCCCCGTGCCTTCCTTGACCCTGGAAGGTGCCACTCCCACTGTCCTTTCCTAATAAAATGAGGAAATTGCATCGCATTGTCTGAGTAGGTGTCATTCTATTCTGGGGGGTGGGGTGGGGCAGGACAGCAAGGGGGAGGATTGGGAAGACAATAGCAGGCATGCTGGGGATGCGGTGGGCTCTATGGCTTCTACTGGGCGGTTTTATGGACAGCAAGCGAACCGGAATTGCCAGCTGGGGCGCCCTCTGGTAAGGTTGGGAAGCCCTGCAAAGTAAACTGGATGGCTTTCTCGCCGCCAAGGATCTGATGGCGCAGGGGATCAAGCTCTGATCAAGAGACAGGATGAGGATCGTTTCGC**ATGATTGAACAAGATGGATTGCACGCAGGTTCTCCGGCCGCTTGGGTGGAGAGGCTATTCGGCTATGACTGGGCACAACAGACAATCGGCTGCTCTGATGCCGCCGTGTTCCGGCTGTCAGCGCAGGGGCGCCCGGTTCTTTTTGTCAAGACCGACCTGTCCGGTGCCCTGAATGAACTGCAAGACGAGGCAGCGCGGCTATCGTGGCTGGCCACGACGGGCGTTCCTTGCGCAGCTGTGCTCGACGTTGTCACTGAAGCGGGAAGGGACTGGCTGCTATTGGGCGAAGTGCCGGGGCAGGATCTCCTGTCATCTCACCTTGCTCCTGCCGAGAAAGTATCCATCATGGCTGATGCAATGCGGCGGCTGCATACGCTTGATCCGGCTACCTGCCCATTCGACCACCAAGCGAAACATCGCATCGAGCGAGCACGTACTCGGATGGAAGCCGGTCTTGTCGATCAGGATGATCTGGACGAAGAGCATCAGGGGCTCGCGCCAGCCGAACTGTTCGCCAGGCTCAAGGCGAGCATGCCCGACGGCGAGGATCTCGTCGTGACCCATGGCGATGCCTGCTTGCCGAATATCATGGTGGAAAATGGCCGCTTTTCTGGATTCATCGACTGTGGCCGGCTGGGTGTGGCGGACCGCTATCAGGACATAGCGTTGGCTACCCGTGATATTGCTGAAGAGCTTGGCGGCGAATGGGCTGACCGCTTCCTCGTGCTTTACGGTATCGCCGCTCCCGATTCGCAGCGCATCGCCTTCTATCGCCTTCTTGACGAGTTCTTCTGA**ATTATTAACGCTTACAATTTCCTGATGCGGTATTTTCTCCTTACGCATCTGTGCGGTATTTCACACCGCATACAGGTGGCACTTTTCGGGGAAATGTGCGCGGAACCCCTATTTGTTTATTTTTCTAAATACATTCAAATATGTATCCGCTCATGAGACAATAACCCTGATAAATGCTTCAATAATAGCACGTGCTAAAACTTCATTTTTAATTTAAAAGGATCTAGGTGAAGATCCTTTTTGATAATCTCATGACCAAAATCCCTTAACGTGAGTTTTCGTTCCACTGAGCGTCAGACCCCGTAGAAAAGATCAAAGGATCTTCTTGAGATCCTTTTTTTCTGCGCGTAATCTGCTGCTTGCAAACAAAAAAACCACCGCTACCAGCGGTGGTTTGTTTGCCGGATCAAGAGCTACCAACTCTTTTTCCGAAGGTAACTGGCTTCAGCAGAGCGCAGATACCAAATACTGTCCTTCTAGTGTAGCCGTAGTTAGGCCACCACTTCAAGAACTCTGTAGCACCGCCTACATACCTCGCTCTGCTAATCCTGTTACCAGTGGCTGCTGCCAGTGGCGATAAGTCGTGTCTTACCGGGTTGGACTCAAGACGATAGTTACCGGATAAGGCGCAGCGGTCGGGCTGAACGGGGGGTTCGTGCACACAGCCCAGCTTGGAGCGAACGACCTACACCGAACTGAGATACCTACAGCGTGAGCTATGAGAAAGCGCCACGCTTCCCGAAGGGAGAAAGGCGGACAGGTATCCGGTAAGCGGCAGGGTCGGAACAGGAGAGCGCACGAGGGAGCTTCCAGGGGGAAACGCCTGGTATCTTTATAGTCCTGTCGGGTTTCGCCACCTCTGACTTGAGCGTCGATTTTTGTGATGCTCGTCAGGGGGGCGGAGCCTATGGAAAAACGCCAGCAACGCGGCCTTTTTACGGTTCCTGGGCTTTTGCTGGCCTTTTGCTCACATGTTCTTGCTGCTTCGCGATGTACGGGCCAGATATACGCGT

**pMA sgRNA backbone**
U6 Promoter
Spacer
Scaffold
Terminator
Backbone

**AGAGGGCCTATTTCCCATGATTCCTTCATATTTGCATATACGATACAAGGCTGTTAGAGAGATAATTAGAATTAATTTGACTGTAAACACAAAGATATTAGTACAAAATACGTGACGTAGAAAGTAATAATTTCTTGGGTAGTTTGCAGTTTTAAAATTATGTTTTAAAATGGACTATCATATGCTTACCGTAACTTGAAAGTATTTCGATTTCTTGGCTTTATATATCTTGTGGAAAGGACGAAACACCGNNNNNNNNNNNNNNNNNNNNGTTTTAGAGCTAGAAATAGCAAGTTAAAATAAGGCTAGTCCGTTATCAACTTGAAAAAGTGGCACCGAGTCGGTGCTTTTTTT**GCCTCATGGGCCTTCCGCTCACTGCCCGCTTTCCAGTCGGGAAACCTGTCGTGCCAGCTGCATTAACATGGTCATAGCTGTTTCCTTGCGTATTGGGCGCTCTCCGCTTCCTCGCTCACTGACTCGCTGCGCTCGGTCGTTCGGGTAAAGCCTGGGGTGCCTAATGAGCAAAAGGCCAGCAAAAGGCCAGGAACCGTAAAAAGGCCGCGTTGCTGGCGTTTTTCCATAGGCTCCGCCCCCCTGACGAGCATCACAAAAATCGACGCTCAAGTCAGAGGTGGCGAAACCCGACAGGACTATAAAGATACCAGGCGTTTCCCCCTGGAAGCTCCCTCGTGCGCTCTCCTGTTCCGACCCTGCCGCTTACCGGATACCTGTCCGCCTTTCTCCCTTCGGGAAGCGTGGCGCTTTCTCATAGCTCACGCTGTAGGTATCTCAGTTCGGTGTAGGTCGTTCGCTCCAAGCTGGGCTGTGTGCACGAACCCCCCGTTCAG

**TLR-Construct**
inaavZNF
Splicing acceptor
T2A
Puromycin
Blasticidin
PolyA
DsRed
Silent GFP
CMV promoter
Backbone

**ATCCTGTCCCTAGGCCACCCACTGTGGGGTCTGACCTCTTCTCTTCCTCCCACAGGGCCTCGAGAGATCTGGCAGCGGAGAGGGCAGAGGAAGTCTTCTAACATGCGGTGACGTGGAGGAGAATCCCGGCCCTAGGCTCGAAATGACCGAGTACAAGCCCACGGTGCGCCTCGCCACCCGCGACGACGTCCCCCGGGCCGTACGCACCCTCGCCGCCGCGTTCGCCGACTACCCCGCCACGCGCCACACCGTCGACCCGGACCGCCACATCGAGCGGGTCACCGAGCTGCAAGAACTCTTCCTCACGCGCGTCGGGCTCGACATCGGCAAGGTGTGGGTCGCGGACGACGGCGCCGCGGTGGCGGTCTGGACCACGCCGGAGAGCGTCGAAGCGGGGGCGGTGTTCGCCGAGATCGGCCCGCGCATGGCCGAGTTGAGCGGTTCCCGGCTGGCCGCGCAGCAACAGATGGAAGGCCTCCTGGCGCCGCACCGGCCCAAGGAGCCCGCGTGGTTCCTGGCCACCGTCGGCGTCTCGCCCGACCACCAGGGCAAGGGTCTGGGCAGCGCCGTCGTGCTCCCCGGAGTGGAGGCGGCCGAGCGCGCCGGGGTGCCCGCCTTCCTGGAGACCTCCGCGCCCCGCAACCTCCCCTTCTACGAGCGGCTCGGCTTCACCGTCACCGCCGACGTCGAGGTGCCCGAAGGACCGCGCACCTGGTGCATGACCCGCAAGCCCGGTGCCCCTTCCTAGACACATCCCTTATGGGAGATCTGGCAGCGGAGAGGGCAGAGGAAGTCTTCTAACATGCGGTGACGTGGAGGAGAATCCCGGCCCTAGGATGGCCAAGCCTTTGTCTCAAGAAGAATCCACCCTCATTGAAAGAGCAACGGCTACAATCAACAGCATCCCCATCTCTGAAGACTACAGCGTCGCCAGCGCAGCTCTCTCTAGCGACGGCCGCATCTTCACTGGTGTCAATGTATATCATTTTACTGGGGGACCTTGTGCAGAACTCGTGGTGCTGGGCACTGCTGCTGCTGCGGCAGCTGGCAACCTGACTTGTATCGTCGCGATCGGAAATGAGAACAGGGGCATCTTGAGCCCCTGCGGACGGTGCCGACAGGTGCTTCTCGATCTGCATCCTGGGATCAAAGCCATAGTGAAGGACAGTGATGGACAGCCGACGGCAGTTGGGATTCGTGAATTGCTGCCCTCTGGTTATGTGTGGGAGGGCTAACGCCCGCCCCACGACCCGCAGCGCCCGACCGAAAGGAGCGCACGACCCCATGGCTCCGACCGAAGCCACCCGGGGCGGCCCCGCCGACCCCGCACCCGCCCCCGAGGCCCACCGACTCTAGAGGGCCCGTTTAAACCCGCTGATCAGCCTCGACTGTGCCTTCTAGTTGCCAGCCATCTGTTGTTTGCCCCTCCCCCGTGCCTTCCTTGACCCTGGAAGGTGCCACTCCCACTGTCCTTTCCTAATAAAATGAGGAAATTGCATCGCATTGTCTGAGTAGGTGTCATTCTATTCTGGGGGGTGGGGTGGGGCAGGACAGCAAGGGGGAGGATTGGGAAGACAATAGCAACCGGTGCTAG**AATTCACTGGCCGTCGTTTTACAACGTCGTGACTGGGAAAACCCTGGCGTTACCCAACTTAATCGCCTTGCAGCACATCCCCCTTTCGCCAGCTGGCGTAATAGCGAAGAGGCCCGCACCGATCGCCCTTCCCAACAGTTGCGCAGCCTGAATGGCGAATGGCGCCTGATGCGGTATTTTCTCCTTACGCATCTGTGCGGTATTTCACACCGCATATGGTGCACTCTCAGTACAATCTGCTCTGATGCCGCATAGTTAAGCCAGCCCCGACACCCGCCAACACCCGCTGACGCGCCCTGACGGGCTTGTCTGCTCCCGGCATCCGCTTACAGACAAGCTGTGACCGTCTCCGGGAGCTGCATGTGTCAGAGGTTTTCACCGTCATCACCGAAACGCGCGAGACGAAAGGGCCTCGTGATACGCCTATTTTTATAGGTTAATGTCATGATAATAATGGTTTCTTAGACGTCAGGTGGCACTTTTCGGGGAAATGTGCGCGGAACCCCTATTTGTTTATTTTTCTAAATACATTCAAATATGTATCCGCTCATGAGACAATAACCCTGATAAATGCTTCAATAATATTGAAAAAGGAAGAGTATGAGTATTCAACATTTCCGTGTCGCCCTTATTCCCTTTTTTGCGGCATTTTGCCTTCCTGTTTTTGCTCACCCAGAAACGCTGGTGAAAGTAAAAGATGCTGAAGATCAGTTGGGTGCACGAGTGGGTTACATCGAACTGGATCTCAACAGCGGTAAGATCCTTGAGAGTTTTCGCCCCGAAGAACGTTTTCCAATGATGAGCACTTTTAAAGTTCTGCTATGTGGCGCGGTATTATCCCGTATTGACGCCGGGCAAGAGCAACTCGGTCGCCGCATACACTATTCTCAGAATGACTTGGTTGAGTACTCACCAGTCACAGAAAAGCATCTTACGGATGGCATGACAGTAAGAGAATTATGCAGTGCTGCCATAACCATGAGTGATAACACTGCGGCCAACTTACTTCTGACAACGATCGGAGGACCGAAGGAGCTAACCGCTTTTTTGCACAACATGGGGGATCATGTAACTCGCCTTGATCGTTGGGAACCGGAGCTGAATGAAGCCATACCAAACGACGAGCGTGACACCACGATGCCTGTAGCAATGGCAACAACGTTGCGCAAACTATTAACTGGCGAACTACTTACTCTAGCTTCCCGGCAACAATTAATAGACTGGATGGAGGCGGATAAAGTTGCAGGACCACTTCTGCGCTCGGCCCTTCCGGCTGGCTGGTTTATTGCTGATAAATCTGGAGCCGGTGAGCGTGGGTCTCGCGGTATCATTGCAGCACTGGGGCCAGATGGTAAGCCCTCCCGTATCGTAGTTATCTACACGACGGGGAGTCAGGCAACTATGGATGAACGAAATAGACAGATCGCTGAGATAGGTGCCTCACTGATTAAGCATTGGTAACTGTCAGACCAAGTTTACTCATATATACTTTAGATTGATTTAAAACTTCATTTTTAATTTAAAAGGATCTAGGTGAAGATCCTTTTTGATAATCTCATGACCAAAATCCCTTAACGTGAGTTTTCGTTCCACTGAGCGTCAGACCCCGTAGAAAAGATCAAAGGATCTTCTTGAGATCCTTTTTTTCTGCGCGTAATCTGCTGCTTGCAAACAAAAAAACCACCGCTACCAGCGGTGGTTTGTTTGCCGGATCAAGAGCTACCAACTCTTTTTCCGAAGGTAACTGGCTTCAGCAGAGCGCAGATACCAAATACTGTTCTTCTAGTGTAGCCGTAGTTAGGCCACCACTTCAAGAACTCTGTAGCACCGCCTACATACCTCGCTCTGCTAATCCTGTTACCAGTGGCTGCTGCCAGTGGCGATAAGTCGTGTCTTACCGGGTTGGACTCAAGACGATAGTTACCGGATAAGGCGCAGCGGTCGGGCTGAACGGGGGGTTCGTGCACACAGCCCAGCTTGGAGCGAACGACCTACACCGAACTGAGATACCTACAGCGTGAGCTATGAGAAAGCGCCACGCTTCCCGAAGGGAGAAAGGCGGACAGGTATCCGGTAAGCGGCAGGGTCGGAACAGGAGAGCGCACGAGGGAGCTTCCAGGGGGAAACGCCTGGTATCTTTATAGTCCTGTCGGGTTTCGCCACCTCTGACTTGAGCGTCGATTTTTGTGATGCTCGTCAGGGGGGCGGAGCCTATGGAAAAACGCCAGCAACGCGGCCTTTTTACGGTTCCTGGCCTTTTGCTGGCCTTTTGCTCACATGTTCTTTCCTGCGTTATCCCCTGATTCTGTGGATAACCGTATTACCGCCTTTGAGTGAGCTGATACCGCTCGCCGCAGCCGAACGACCGAGCGCAGCGAGTCAGTGAGCGAGGAAGCGGAAGAGCGCCCAATACGCAAACCGCCTCTCCCCGCGCGTTGGCCGATTCATTAATGCAGCTGGCACGACAGGTTTCCCGACTGGAAAGCGGGCAGTGAGCGCAACGCAATTAATGTGAGTTAGCTCACTCATTAGGCACCCCAGGCTTTACACTTTATGCTTCCGGCTCGTATGTTGTGTGGAATTGTGAGCGGATAACAATTTCACACAGGAAACAGCTATGACCATGATTACGCCAAGCTTCTTAAGTAGACGCGTCCATAGAGCCCACCGCATCCCCAGCATGCCGATTT**TACCACATTTGTAGAGGTTTTACTTGCTTTAAAAAACCTCCCACACCTCCCCCTGAACCTGAAACATAAAATGAATGCAATTGTTGTTGTTAACTTGTTTATTGCAGCTTATAATGGTTACAAATAAAGCAATAGCATCACAAATTTCACAAATAAAGCATTTTTTTCACTGCATTCTAGTTGTGGTTTGTCCAAACTCATCAATGTATCTTATCATGTCTGCTCGAAGCGGCCGCTACAGGAACAGGTGGTGGCGGCCCTCGGCGCGCTCGTACTGCTCCACGATGGTGTAGTCCTCGTTGTGGGAGGTGATGTCCAGCTTGGAGTCCACGTAGTAGTAGCCGGGCAGCTGCACGGGCTTCTTGGCCATGTAGATGGACTTGAACTCCACCAGGTAGTGGCCGCCGTCCTTCAGCTTCAGGGCCTTGTGGATCTCGCCCTTCAGCACGCCGTCGCGGGGGTACAGGCGCTCGGTGGAGGCCTCCCAGCCCATAGTCTTCTTCTGCATTACGGGGCCGTCGGAGGGGAAGTTCACGCCGATGAACTTCACCTTGTAGATGAAGGAGCCGTCCTGCAGGGAGGAGTCCTGGGTCACGGTCACCACGCCGCCGTCCTCGAAGTTCATCACGCGCTCCCACTTGAAGCCCTCGGGGAAGGACAGCTTCTTGTAGTCGGGGATGTCGGCGGGGTGCTTCACGTACACCTTGGAGCCGTACTGGAACTGGGGGGACAGGATGTCCCAGGCGAAGGGCAGGGGGCCGCCCTTGGTCACCTTCAGCTTGGCGGTCTGGGTGCCCTCGTAGGGGCGGCCCTCGCCCTCGCCCTCGATCTCGAACTCGTGGCCGTTCACGGAGCCCTCCATGCGCACCTTGAAGCGCATGAACTCCTTGATGACGTCCTCGGAGGAGGCAGGGCCCGGATTCTCCTCCACGTCACCGCATGTTAGAAGACTTCCTCTGCCCTCACCGGGAGCTCTTACTTGTACAGCTCGTCCATGCCGAGAGTGATCCCGGCGGCGGTCACGAACTCCAGCAGGACCATGTGATCGCGCTTCTCGTTGGGGTCTTTGCTCAGGGCGGACTGGGTGCTCAGGTAGTGGTTGTCGGGCAGCAGCACGGGGCCGTCGCCAATAGGTGTGTTCTGCTGGTAGTGGTCGGCGAGCTGCACGCTGCCGTCCTCGATGTTGTGGCGGATCTTGAAGTTCACCTTGATGCCGTTCTTCTGCTTGTCGGCCATGATATAGACGTTGTGGCTGTTGTAGTTGTACTCCAGCTTGTGCCCCAGGATGTTGCCGTCCTCCTTGAAGTCGATGCCCTTCAGCTCGATGCGGTTCACCAGGGTGTCGCCCTCGAACTTCACCTCGGCGCGGGTCTTGTAGTTGCCGTCGTCCTTGAAGAAGATGGTGCGCTCCTGGACGTAGCCTTCGGGCATGGCGGACTTGAAGAAGTCGTGCTGCTTCATGTGGTCGGGGTAGCGGCTGAAGCACTGCACGCATCTGCGGCCAGGGTGGTCACGAGGGTGGGCCAGGGCACGGGCAGCTTGCCGGTGGTGCAGATGAACTTCAGGGTCAGCTTGCCGTAGGTGGCATCGCCCTCGCCCTCGCCGGACACGCTGAACTTGTGGCCGTTTACGTCGCCGTCCAGCTCGACCAGGATGGGCACCACCCCGGTGAACAGCTCCTCGCCCTTGCTCACCAT**GGTGGCTCTCGAGAGCGGATCTGACGGTTCACTAAACCAGCTCTGCTTATATAGACCTCCCACCGTACACGCCTACCGCCCATTTGCGTCAATGGGGCGGAGTTGTTACGACATTTTGGAAAGTCCCGTTGATTTTGGTGCCAAAACAAACTCCCATTGACGTCAATGGGGTGGAGACTTGGAAATCCCCGTGAGTCAAACCGCTATCCACGCCCATTGATGTACTGCCAAAACCGCATCACCATGGTAATAGCGATGACTAATACGTAGATGTACTGCCAAGTAGGAAAGTCCCATAAGGTCATGTACTGGGCATAATGCCAGGCGGGCCATTTACCGTCATTGACGTCAATAGGGGGCGTACTTGGCATATGATACACTTGATGTACTGCCAAGTGGGCAGTTTACCGTAAATACTCCACCCATTGACGTCAATGGAAAGTCCCTATTGGCGTTACTATGGGAACATACGTCATTATTGACGTCAATGGGCGGGGGTCGTTGGGCGGTCAGCCAGGCGGGCCATTTACCGTAAGTTATGTAACGCGGAACTCCATATATGGGCTATGAACTAATGACCCCGTAATTGATTACTATTAATAACTAACGCGTGATCTCGAGGAATTCGAGCTCGGTACCTCGCGAATGCATCTAGATATCACGCGT

**SpCas9-TLRsgRNA**

U6 promoter
Spacer
Scaffold
CMV promoter
SpCas9
T2A
Puromycin
Bovine Growth Hormone Polyadenylation (BGH-PolyA) signal

GACTCTTCGCGATGTACGGGCCAGATATACGCGTTGTACAAAAAAGCAGGCTTTAAAGGAACCAATTCAGTCGACTGGATCCGGTACCAAGGTCGGGCAGGA**AGAGGGCCTATTTCCCATGATTCCTTCATATTTGCATATACGATACAAGGCTGTTAGAGAGATAATTAGAATTAATTTGACTGTAAACACAAAGATATTAGTACAAAATACGTGACGTAGAAAGTAATAATTTCTTGGGTAGTTTGCAGTTTTAAAATTATGTTTTAAAATGGACTATCATATGCTTACCGTAACTTGAAAGTATTTCGATTTCTTGGCTTTATATATCTTGTGGAAAGGACGAAACACC**GGACTGCACGCATCTGCGGCCA**GTTTTAGAGCTAGAAATAGCAAGTTAAAATAAGGCTAGTCCGTTATCAACTTGAAAAAGTGGCACCGAGTCGGTGC**TTTTTTTCTAGACCCAGCTTTCTTGTACAAAGTTGGCATTAA**CGCGTTGACATTGATTATTGACTAGTTATTAATAGTAATCAATTACGGGGTCATTAGTTCATAGCCCATATATGGAGTTCCGCGTTACATAACTTACGGTAAATGGCCCGCCTGGCTGACCGCCCAACGACCCCCGCCCATTGACGTCAATAATGACGTATGTTCCCATAGTAACGCCAATAGGGACTTTCCATTGACGTCAATGGGTGGACTATTTACGGTAAACTGCCCACTTGGCAGTACATCAAGTGTATCATATGCCAAGTACGCCCCCTATTGACGTCAATGACGGTAAATGGCCCGCCTGGCATTATGCCCAGTACATGACCTTATGGGACTTTCCTACTTGGCAGTACATCTACGTATTAGTCATCGCTATTACCATGGTGATGCGGTTTTGGCAGTACATCAATGGGCGTGGATAGCGGTTTGACTCACGGGGATTTCCAAGTCTCCACCCCATTGACGTCAATGGGAGTTTGTTTTGGCACCAAAATCAACGGGACTTTCCAAAATGTCGTAACAACTCCGCCCCATTGACGCAAATGGGCGGTAGGCGTGTACGGTGGGAGGTCTATATAAGCAGAGCTC**TCTGGCTAACTAGAGAACCCACTGCTTACTGGCTTATCGAAATTAATACGACTCACTATAGGGAGACCCAAGCTGGCTAGCGTTTAAACTTAAGCTGATCCACTAGTCCAGTGTGGTGGAATTCGCC**ATGGCTCCTAAGAAAAAGCGGAAGGTGGACAAGAAATACTCAATCGGGCTGGACATCGGAACTAACTCAGTGGGGTGGGCAGTCATTACTGACGAGTACAAAGTGCCAAGCAAGAAATTTAAGGTCCTGGGCAACACCGATAGGCACTCCATCAAGAAAAATCTGATTGGGGCCCTGCTGTTCGACTCTGGAGAGACAGCTGAAGCAACTAGACTGAAAAGGACTGCTAGAAGGCGCTATACCCGGCGAAAGAATCGCATCTGCTACCTGCAGGAGATTTTCTCTAACGAAATGGCCAAGGTGGACGATAGTTTCTTTCATCGGCTGGAGGAATCATTCCTGGTCGAGGAAGATAAGAAACACGAGAGACATCCTATCTTTGGAAACATTGTGGACGAGGTCGCTTATCACGAAAAATACCCCACCATCTATCATCTGCGCAAGAAACTGGTGGACTCTACAGATAAAGCAGACCTGCGGCTGATCTATCTGGCCCTGGCTCACATGATTAAGTTCAGAGGCCATTTTCTGATCGAGGGAGATCTGAACCCAGACAATAGCGATGTGGACAAGCTGTTCATCCAGCTGGTCCAGACATACAATCAGCTGTTTGAGGAAAACCCTATTAATGCATCTGGCGTGGACGCAAAAGCCATCCTGAGTGCCAGGCTGTCTAAGAGTAGAAGGCTGGAGAACCTGATCGCTCAGCTGCCAGGCGAAAAGAAAAACGGCCTGTTTGGAAATCTGATTGCACTGTCACTGGGACTGACACCTAACTTCAAGAGCAATTTTGATCTGGCCGAGGACGCTAAACTGCAGCTGAGCAAGGACACTTATGACGATGACCTGGATAACCTGCTGGCTCAGATCGGAGATCAGTACGCAGACCTGTTCCTGGCCGCTAAGAATCTGTCTGACGCTATCCTGCTGAGTGATATTCTGCGGGTGAACACCGAGATTACAAAAGCCCCTCTGTCAGCTAGCATGATCAAGAGATATGACGAGCACCATCAGGATCTGACCCTGCTGAAGGCACTGGTGCGCCAGCAGCTGCCCGAGAAGTACAAGGAAATCTTCTTTGATCAGAGTAAGAACGGGTACGCCGGTTATATTGACGGCGGAGCTTCACAGGAGGAATTCTACAAGTTTATCAAACCTATTCTGGAGAAGATGGACGGCACCGAGGAACTGCTGGTGAAACTGAATCGCGAGGACCTGCTGCGCAAGCAGCGGACATTTGATAACGGCTCCATCCCCCACCAGATTCATCTGGGAGAGCTGCACGCAATCCTGCGACGACAGGAAGACTTCTACCCATTTCTGAAGGATAACCGCGAGAAGATCGAAAAAATTCTGACCTTCCGGATCCCTTACTATGTGGGGCCCCTGGCAAGGGGTAATTCCCGCTTTGCCTGGATGACACGGAAATCTGAGGAAACAATCACTCCTTGGAACTTCGAGGAAGTGGTCGATAAGGGAGCTTCCGCACAGTCTTTCATCGAGAGAATGACAAACTTCGACAAAAACCTGCCAAATGAGAAAGTGCTGCCTAAGCACAGTCTGCTGTACGAGTATTTCACAGTCTATAACGAACTGACTAAGGTGAAATACGTCACCGAGGGGATGAGGAAGCCCGCCTTCCTGAGCGGTGAACAGAAGAAAGCTATCGTGGACCTGCTGTTTAAAACCAATCGCAAGGTGACAGTCAAGCAGCTGAAGGAGGACTACTTCAAGAAAATTGAATGTTTCGATTCTGTGGAGATCAGTGGCGTCGAAGACAGATTTAACGCTTCTCTGGGAACCTACCACGATCTGCTGAAGATCATTAAGGATAAAGACTTCCTGGACAACGAGGAAAATGAGGATATCCTGGAAGACATTGTGCTGACCCTGACACTGTTTGAGGATCGCGAAATGATCGAGGAACGGCTGAAAACTTATGCCCATCTGTTCGATGACAAGGTGATGAAACAGCTGAAGCGAAGAAGGTACACCGGCTGGGGACGACTGAGCAGAAAGCTGATCAACGGCATTCGGGACAAACAGAGTGGAAAGACTATCCTGGACTTTCTGAAATCAGATGGCTTCGCTAACAGAAATTTTATGCAGCTGATTCACGATGACAGCCTGACCTTCAAAGAGGATATCCAGAAGGCACAGGTGTCCGGGCAGGGTGACTCTCTGCACGAGCATATCGCAAACCTGGCCGGGTCCCCCGCCATCAAGAAAGGTATTCTGCAGACCGTGAAGGTGGTCGATGAGCTGGTGAAAGTCATGGGCAGGCATAAGCCAGAAAACATCGTGATTGAGATGGCCCGCGAAAATCAGACCACACAGAAAGGACAGAAGAACAGCCGCGAGCGGATGAAAAGGATCGAGGAAGGCATTAAGGAACTGGGATCCCAGATCCTGAAAGAGCACCCTGTGGAAAACACTCAGCTGCAGAATGAGAAGCTGTATCTGTACTATCTGCAGAATGGGCGGGATATGTACGTGGACCAGGAGCTGGATATTAACCGACTGTCTGATTACGACGTGGATCATATCGTCCCACAGTCATTCCTGAAAGATGACAGCATTGACAATAAGGTGCTGACCCGGAGTGACAAAAACCGAGGAAAGAGTGATAATGTCCCTTCAGAGGAAGTGGTCAAGAAAATGAAGAACTACTGGAGACAGCTGCTGAATGCCAAACTGATCACACAGCGAAAGTTTGATAACCTGACTAAAGCTGAGAGAGGGGGTCTGTCAGAACTGGACAAAGCAGGCTTCATCAAGCGACAGCTGGTGGAGACCAGACAGATCACAAAGCACGTCGCTCAGATTCTGGATAGCAGGATGAACACAAAGTACGATGAGAATGACAAACTGATCCGCGAAGTGAAGGTCATTACTCTGAAGTCAAAACTTGTGAGCGACTTCAGAAAGGATTTCCAGTTCTACAAAGTCAGGGAGATCAACAATTATCACCATGCTCATGACGCATACCTGAACGCAGTGGTCGGGACCGCCCTGATTAAGAAATACCCCAAACTGGAGAGCGAATTCGTGTACGGTGACTATAAGGTGTACGATGTCAGAAAAATGATCGCCAAGAGTGAGCAGGAAATTGGAAAAGCCACCGCTAAGTATTTCTTTTACTCAAACATCATGAATTTCTTTAAGACTGAGATCACCCTGGCAAATGGGGAAATCCGAAAGAGACCACTGATTGAGACTAACGGCGAGACCGGAGAAATCGTGTGGGACAAGGGTAGGGATTTTGCCACAGTGCGCAAGGTCCTGTCCATGCCTCAAGTGAATATTGTCAAGAAAACAGAGGTGCAGACTGGCGGATTCAGTAAGGAATCAATTCTGCCCAAACGGAACTCTGATAAGCTGATCGCCCGAAAGAAAGACTGGGATCCCAAGAAATATGGGGGTTTCGACTCCCCAACAGTGGCTTACTCTGTCCTGGTGGTCGCAAAGGTGGAGAAGGGGAAAAGCAAGAAACTGAAATCCGTCAAGGAGCTGCTGGGTATCACTATTATGGAGAGGAGCTCCTTCGAGAAGAACCCCATCGATTTTCTGGAGGCTAAAGGCTATAAGGAAGTGAAGAAAGACCTGATCATTAAACTGCCAAAGTACAGCCTGTTTGAGCTGGAAAACGGAAGGAAGCGAATGCTGGCATCCGCAGGAGAGCTGCAGAAGGGTAATGAACTGGCCCTGCCTTCTAAGTACGTGAACTTCCTGTATCTGGCTAGCCACTACGAGAAGCTGAAAGGCTCCCCCGAGGATAACGAACAGAAACAGCTGTTTGTGGAGCAGCACAAGCATTATCTGGACGAGATCATTGAACAGATTAGCGAGTTCTCCAAAAGAGTGATCCTGGCTGACGCAAATCTGGATAAGGTCCTGAGCGCATACAACAAACACAGAGATAAGCCAATCAGGGAGCAGGCCGAAAATATCATTCATCTGTTCACTCTGACCAACCTGGGAGCCCCTGCAGCCTTCAAGTATTTTGACACTACCATCGATCGGAAACGATACACATCCACTAAGGAGGTGCTGGACGCTACCCTGATTCACCAGAGCATTACCGGCCTGTATGAAACAAGGATTGACCTGTCTCAGCTGGGGGGCGAC**CTCGAGAGATCT**GGCAGCGGAGAGGGCAGAGGAAGTCTTCTAACATGCGGTGACGTGGAGGAGAATCCCGGCCCT**AGGCTCGAAATGACCGAGTACAAGCCCACGGTGCGCCTCGCCACCCGCGACGACGTCCCCCGGGCCGTACGCACCCTCGCCGCCGCGTTCGCCGACTACCCCGCCACGCGCCACACCGTCGACCCGGACCGCCACATCGAGCGGGTCACCGAGCTGCAAGAACTCTTCCTCACGCGCGTCGGGCTCGACATCGGCAAGGTGTGGGTCGCGGACGACGGCGCCGCGGTGGCGGTCTGGACCACGCCGGAGAGCGTCGAAGCGGGGGCGGTGTTCGCCGAGATCGGCCCGCGCATGGCCGAGTTGAGCGGTTCCCGGCTGGCCGCGCAGCAACAGATGGAAGGCCTCCTGGCGCCGCACCGGCCCAAGGAGCCCGCGTGGTTCCTGGCCACCGTCGGCGTCTCGCCCGACCACCAGGGCAAGGGTCTGGGCAGCGCCGTCGTGCTCCCCGGAGTGGAGGCGGCCGAGCGCGCCGGGGTGCCCGCCTTCCTGGAGACCTCCGCGCCCCGCAACCTCCCCTTCTACGAGCGGCTCGGCTTCACCGTCACCGCCGACGTCGAGGTGCCCGAAGGACCGCGCACCTGGTGCATGACCCGCAAGCCCGGTGCCTGACGCCCGCCCCACTACCCGCAGCGCCCGACCGAAAGGAGCGCACGACCCCATGGCTCCGACCGAAGCCACCCGGGGCGGCCCCGCCGACCCCGCACCCGCCCCCGAGGCCCACCGACTCTAGAGGGCCCGTTTAAACCCGCTGATCAGCCTCGA**CTGTGCCTTCTAGTTGCCAGCCATCTGTTGTTTGCCCCTCCCCCGTGCCTTCCTTGACCCTGGAAGGTGCCACTCCCACTGTCCTTTCCTAATAAAATGAGGAAATTGCATCGCATTGTCTGAGTAGGTGTCATTCTATTCTGGGGGGTGGGGTGGGGCAGGACAGCAAGGGGGAGGATTGGGAAGACAATAGCAGGCATGCTGGGGATGCGGTGGGCTCTATGG**CTTCTACTGGGCGGTTTTATGGACAGCAAGCGAACCGGAATTGCCAGCTGGGGCGCCCTCTGGTAAGGTTGGGAAGCCCTGCAAAGTAAACTGGATGGCTTTCTCGCCGCCAAGGATCTGATGGCGCAGGGGATCAAGCTCTGATCAAGAGACAGGATGAGGATCGTTTCGCATGATTGAACAAGATGGATTGCACGCAGGTTCTCCGGCCGCTTGGGTGGAGAGGCTATTCGGCTATGACTGGGCACAACAGACAATCGGCTGCTCTGATGCCGCCGTGTTCCGGCTGTCAGCGCAGGGGCGCCCGGTTCTTTTTGTCAAGACCGACCTGTCCGGTGCCCTGAATGAACTGCAAGACGAGGCAGCGCGGCTATCGTGGCTGGCCACGACGGGCGTTCCTTGCGCAGCTGTGCTCGACGTTGTCACTGAAGCGGGAAGGGACTGGCTGCTATTGGGCGAAGTGCCGGGGCAGGATCTCCTGTCATCTCACCTTGCTCCTGCCGAGAAAGTATCCATCATGGCTGATGCAATGCGGCGGCTGCATACGCTTGATCCGGCTACCTGCCCATTCGACCACCAAGCGAAACATCGCATCGAGCGAGCACGTACTCGGATGGAAGCCGGTCTTGTCGATCAGGATGATCTGGACGAAGAGCATCAGGGGCTCGCGCCAGCCGAACTGTTCGCCAGGCTCAAGGCGAGCATGCCCGACGGCGAGGATCTCGTCGTGACCCATGGCGATGCCTGCTTGCCGAATATCATGGTGGAAAATGGCCGCTTTTCTGGATTCATCGACTGTGGCCGGCTGGGTGTGGCGGACCGCTATCAGGACATAGCGTTGGCTACCCGTGATATTGCTGAAGAGCTTGGCGGCGAATGGGCTGACCGCTTCCTCGTGCTTTACGGTATCGCCGCTCCCGATTCGCAGCGCATCGCCTTCTATCGCCTTCTTGACGAGTTCTTCTGAATTATTAACGCTTACAATTTCCTGATGCGGTATTTTCTCCTTACGCATCTGTGCGGTATTTCACACCGCATACAGGTGGCACTTTTCGGGGAAATGTGCGCGGAACCCCTATTTGTTTATTTTTCTAAATACATTCAAATATGTATCCGCTCATGAGACAATAACCCTGATAAATGCTTCAATAATAGCACGTGCTAAAACTTCATTTTTAATTTAAAAGGATCTAGGTGAAGATCCTTTTTGATAATCTCATGACCAAAATCCCTTAACGTGAGTTTTCGTTCCACTGAGCGTCAGACCCCGTAGAAAAGATCAAAGGATCTTCTTGAGATCCTTTTTTTCTGCGCGTAATCTGCTGCTTGCAAACAAAAAAACCACCGCTACCAGCGGTGGTTTGTTTGCCGGATCAAGAGCTACCAACTCTTTTTCCGAAGGTAACTGGCTTCAGCAGAGCGCAGATACCAAATACTGTCCTTCTAGTGTAGCCGTAGTTAGGCCACCACTTCAAGAACTCTGTAGCACCGCCTACATACCTCGCTCTGCTAATCCTGTTACCAGTGGCTGCTGCCAGTGGCGATAAGTCGTGTCTTACCGGGTTGGACTCAAGACGATAGTTACCGGATAAGGCGCAGCGGTCGGGCTGAACGGGGGGTTCGTGCACACAGCCCAGCTTGGAGCGAACGACCTACACCGAACTGAGATACCTACAGCGTGAGCTATGAGAAAGCGCCACGCTTCCCGAAGGGAGAAAGGCGGACAGGTATCCGGTAAGCGGCAGGGTCGGAACAGGAGAGCGCACGAGGGAGCTTCCAGGGGGAAACGCCTGGTATCTTTATAGTCCTGTCGGGTTTCGCCACCTCTGACTTGAGCGTCGATTTTTGTGATGCTCGTCAGGGGGGCGGAGCCTATGGAAAAACGCCAGCAACGCGGCCTTTTTACGGTTCCTGGGCTTTTGCTGGCCTTTTGCTCACATGTTCTT

**TLR-GFP-Donor**

Homology arms

Insert

GGGGAATTGTGAGCGGATAACAATTCCCCTGTAGAAATAATTTTGTTTAACTTTAATAAGGAGATATACCATGGGCAGCAGCCATCACCATCATCACCACAGCCAGGATCC**GAGCAAGGGCGAGGAGCTGTTCACCGGGGTGGTGCCCATCCTGGTCGAGCTGGACGGCGACGTAAACGGCCACAAGTTCAGCGTGTCCGGCGAGGGCGAGGGCGATGCCACCTACGGCAAGCTGACCCTGAAGTTCATCTGCACCACCGGCAAGCTGCCCGTGCCCTGGCCCACCCTCGTGACCACCCTGACCTACGGCGTGCAGTGCTTCAGCCGCTACCCCGACCACATGAAGCAGCACGACTTCTTCAAGTCCGCCATGCCCGAAGGCTACGTCCAGGAGCGCACCATCTTCTTCAAGGACGACGGCAACTACAAGACCCGCGCCGAGGTGAAGTTCGAGGGCGACACCCTGGTGAACCGCATCGAGCTGAAGGGCATCGACTTCAAGGAGGACGGCAACATCCTGGGGCACAAGCTGGAGTACAACTACAACAGCCACAACGTCTATATCATGGCCGACAAGCAGAAGAACGGCATCAAGGTGAACTTCAAGATCCGCCACAACATCGAGGACGGCAGCGTGCAGCTCGCCGACCACTACCAGCAGAACACCCCCATCGGCGACGGCCCCGTGCTGCTGCCCGACAACCACTACCTGAGCACCCAGTCCGCCCTGAGCAAAGACCCCAACGAGAAGCGCGATCACATGGTCCTGCTGGAGTTCGTGACCGCCGCCGGGATCACTCTCGGCATGGACGAGCTGTACAAG**TAAGCGGCCGCATAATGCTTAAGTCGAACAGAAAGTAATCGTATTGTACACGGCCGCATAATCGAAATTAATACGACTCACTATAGGGGAATTGTGAGCGGATAACAATTCCCCATCTTAGTATATTAGTTAAGTATAAGAAGGAGATATACATATGGCAGATCTCAATTGGATATCGGCCGGCCACGCGATCGCTGACGTCGGTACCCTCGAGTCTGGTAAAGAAACCGCTGCTGCGAAATTTGAACGCCAGCACATGGACTCGTCTACTAGCGCAGCTTAATTAACCTAGGCTGCTGCCACCGCTGAGCAATAACTAGCATAACCCCTTGGGGCCTCTAAACGGGTCTTGAGGGGTTTTTTGCTGAAACCTCAGGCATTTGAGAAGCACACGGTCACACTGCTTCCGGTAGTCAATAAACCGGTAAACCAGCAATAGACATAAGCGGCTATTTAACGACCCTGCCCTGAACCGACGACCGGGTCGAATTTGCTTTCGAATTTCTGCCATTCATCCGCTTATTATCACTTATTCAGGCGTAGCACCAGGCGTTTAAGGGCACCAATAACTGCCTTAAAAAAATTACGCCCCGCCCTGCCACTCATCGCAGTACTGTTGTAATTCATTAAGCATTCTGCCGACATGGAAGCCATCACAGACGGCATGATGAACCTGAATCGCCAGCGGCATCAGCACCTTGTCGCCTTGCGTATAATATTTGCCCATAGTGAAAACGGGGGCGAAGAAGTTGTCCATATTGGCCACGTTTAAATCAAAACTGGTGAAACTCACCCAGGGATTGGCTGAGACGAAAAACATATTCTCAATAAACCCTTTAGGGAAATAGGCCAGGTTTTCACCGTAACACGCCACATCTTGCGAATATATGTGTAGAAACTGCCGGAAATCGTCGTGGTATTCACTCCAGAGCGATGAAAACGTTTCAGTTTGCTCATGGAAAACGGTGTAACAAGGGTGAACACTATCCCATATCACCAGCTCACCGTCTTTCATTGCCATACGGAACTCCGGATGAGCATTCATCAGGCGGGCAAGAATGTGAATAAAGGCCGGATAAAACTTGTGCTTATTTTTCTTTACGGTCTTTAAAAAGGCCGTAATATCCAGCTGAACGGTCTGGTTATAGGTACATTGAGCAACTGACTGAAATGCCTCAAAATGTTCTTTACGATGCCATTGGGATATATCAACGGTGGTATATCCAGTGATTTTTTTCTCCATTTTAGCTTCCTTAGCTCCTGAAAATCTCGATAACTCAAAAAATACGCCCGGTAGTGATCTTATTTCATTATGGTGAAAGTTGGAACCTCTTACGTGCCGATCAACGTCTCATTTTCGCCAAAAGTTGGCCCAGGGCTTCCCGGTATCAACAGGGACACCAGGATTTATTTATTCTGCGAAGTGATCTTCCGTCACAGGTATTTATTCGGCGCAAAGTGCGTCGGGTGATGCTGCCAACTTACTGATTTAGTGTATGATGGTGTTTTTGAGGTGCTCCAGTGGCTTCTGTTTCTATCAGCTGTCCCTCCTGTTCAGCTACTGACGGGGTGGTGCGTAACGGCAAAAGCACCGCCGGACATCAGCGCTAGCGGAGTGTATACTGGCTTACTATGTTGGCACTGATGAGGGTGTCAGTGAAGTGCTTCATGTGGCAGGAGAAAAAAGGCTGCACCGGTGCGTCAGCAGAATATGTGATACAGGATATATTCCGCTTCCTCGCTCACTGACTCGCTACGCTCGGTCGTTCGACTGCGGCGAGCGGAAATGGCTTACGAACGGGGCGGAGATTTCCTGGAAGATGCCAGGAAGATACTTAACAGGGAAGTGAGAGGGCCGCGGCAAAGCCGTTTTTCCATAGGCTCCGCCCCCCTGACAAGCATCACGAAATCTGACGCTCAAATCAGTGGTGGCGAAACCCGACAGGACTATAAAGATACCAGGCGTTTCCCCTGGCGGCTCCCTCGTGCGCTCTCCTGTTCCTGCCTTTCGGTTTACCGGTGTCATTCCGCTGTTATGGCCGCGTTTGTCTCATTCCACGCCTGACACTCAGTTCCGGGTAGGCAGTTCGCTCCAAGCTGGACTGTATGCACGAACCCCCCGTTCAGTCCGACCGCTGCGCCTTATCCGGTAACTATCGTCTTGAGTCCAACCCGGAAAGACATGCAAAAGCACCACTGGCAGCAGCCACTGGTAATTGATTTAGAGGAGTTAGTCTTGAAGTCATGCGCCGGTTAAGGCTAAACTGAAAGGACAAGTTTTGGTGACTGCGCTCCTCCAAGCCAGTTACCTCGGTTCAAAGAGTTGGTAGCTCAGAGAACCTTCGAAAAACCGCCCTGCAAGGCGGTTTTTTCGTTTTCAGAGCAAGAGATTACGCGCAGACCAAAACGATCTCAAGAAGATCATCTTATTAATCAGATAAAATATTTCTAGATTTCAGTGCAATTTATCTCTTCAAATGTAGCACCTGAAGTCAGCCCCATACGATATAAGTTGTAATTCTCATGTTAGTCATGCCCCGCGCCCACCGGAAGGAGCTGACTGGGTTGAAGGCTCTCAAGGGCATCGGTCGAGATCCCGGTGCCTAATGAGTGAGCTAACTTACATTAATTGCGTTGCGCTCACTGCCCGCTTTCCAGTCGGGAAACCTGTCGTGCCAGCTGCATTAATGAATCGGCCAACGCGCGGGGAGAGGCGGTTTGCGTATTGGGCGCCAGGGTGGTTTTTCTTTTCACCAGTGAGACGGGCAACAGCTGATTGCCCTTCACCGCCTGGCCCTGAGAGAGTTGCAGCAAGCGGTCCACGCTGGTTTGCCCCAGCAGGCGAAAATCCTGTTTGATGGTGGTTAACGGCGGGATATAACATGAGCTGTCTTCGGTATCGTCGTATCCCACTACCGAGATGTCCGCACCAACGCGCAGCCCGGACTCGGTAATGGCGCGCATTGCGCCCAGCGCCATCTGATCGTTGGCAACCAGCATCGCAGTGGGAACGATGCCCTCATTCAGCATTTGCATGGTTTGTTGAAAACCGGACATGGCACTCCAGTCGCCTTCCCGTTCCGCTATCGGCTGAATTTGATTGCGAGTGAGATATTTATGCCAGCCAGCCAGACGCAGACGCGCCGAGACAGAACTTAATGGGCCCGCTAACAGCGCGATTTGCTGGTGACCCAATGCGACCAGATGCTCCACGCCCAGTCGCGTACCGTCTTCATGGGAGAAAATAATACTGTTGATGGGTGTCTGGTCAGAGACATCAAGAAATAACGCCGGAACATTAGTGCAGGCAGCTTCCACAGCAATGGCATCCTGGTCATCCAGCGGATAGTTAATGATCAGCCCACTGACGCGTTGCGCGAGAAGATTGTGCACCGCCGCTTTACAGGCTTCGACGCCGCTTCGTTCTACCATCGACACCACCACGCTGGCACCCAGTTGATCGGCGCGAGATTTAATCGCCGCGACAATTTGCGACGGCGCGTGCAGGGCCAGACTGGAGGTGGCAACGCCAATCAGCAACGACTGTTTGCCCGCCAGTTGTTGTGCCACGCGGTTGGGAATGTAATTCAGCTCCGCCATCGCCGCTTCCACTTTTTCCCGCGTTTTCGCAGAAACGTGGCTGGCCTGGTTCACCACGCGGGAAACGGTCTGATAAGAGACACCGGCATACTCTGCGACATCGTATAACGTTACTGGTTTCACATTCACCACCCTGAATTGACTCTCTTCCGGGCGCTATCATGCCATACCGCGAAAGGTTTTGCGCCATTCGATGGTGTCCGGGATCTCGACGCTCTCCCTTATGCGACTCCTGCATTAGGAAATTAATACGACTCACTATA

**HBEGF-HDR-Donor-plasmid**

Homology arms

Insert

**GAGGGAGAAGCCAGTAGGAAACATGGCAGAGTGGGCTTCCAGGGCAGAGTAGAGCTCCTGTGGGAAGGTAGGAAGTGCATTTGGATGCATGATGTATAGGTATGTGTGTATTTGGGTTTATGTGCATGTAAGTGTGCAAATGTGGATTGACTGTGAGGCATGGCAGGACTGTACAGAGAGGGATCATCATGGCGGCAGGTTGAGGCCTCTCTTTCTTCTTCCTTATCCCAGCAAGGACGAGGAGGTGGGAGACATGGAGAGTACTGGCCTTTGGCCACGTTGTGAGAGAACAATTCCTTTGTGCAGGGTTCACAGGAAATGGAACCTGACCCATTAGGCATCAGCCCCCGCAATTGGTCAGGCAACATCACCCCTTCCCTGGGTAGGTGTGTGGGTGGAGGGGCTGTGGGTTCCTTAGCCTCTCTCCTAAGCCAAACCCAGCAAACGGCTGCCTTGGCAACCCCTCAGGGATGACAGCACTGCCATGCTCTCTGGCAGGCATAATGTTGCCACTGTGCCTGAGGCCAACACCCTGCGTCAGGCTGCAAACATCCATTCCCTTCCCTGTGGGGAGGGAGGCTCTGGGGGCCTTAGTGGGAGACTCTGGACAGGGCCAAGAGACTGTTGTATGCACACTGCCTCCAGCCTGTCAAGAAGGCGGCGTGCCTGGCATCCCTTCTACTGGTGATTGGTGCAGATCCCTTA**G

**HDRT-plasmid**

Homology arms
Splicing acceptor
T2A
GFP
PolyA
Ampicillin resistance
Backbone

CCTGCAGGCAGCTGCGCGCTCGCTCGCTCACTGAGGCCGCCCGGGCGTCGGGCGACCTTTGGTCGCCCGGCCTCAGTGAGCGAGCGAGCGCGCAGAGAGGGAGTGGCCAACTCCATCACTAGGGGTTCCTGCGGCCGCATCGATTGAATTCCCCGGGGATCCTCTAGAGTCCTCAGCGCTCTTCAGCTG**TGAAATCATGGCCTCTTGGCCAAGATTGATAGCTTGTGCCTGTCCCTGAGTCCCAGTCCATCACGAGCAGCTGGTTTCTAAGATGCTATTTCCCGTATAAAGCATGAGACCGTGACTTGCCAGCCCCACAGAGCCCCGCCCTTGTCCATCACTGGCATCTGGACTCCAGCCTGGGTTGGGGCAAAGAGGGAAATGAGATCATGTCCTAACCCTGATCCTCTTGT**AAGCTT**CTGACCTCTTCTCTTCCTCCCACAG**GGCCTCGAGCT**GGCAGCGGAGAGGGCAGAGGAAGTCTTCTAACATGCGGTGACGTGGAGGAGAATCCCGGCCCT**AGGCTCGAG**ATGGTGAGCAAGGGCGAGGAGCTGTTCACCGGGGTGGTGCCCATCCTGGTCGAGCTGGACGGCGACGTAAACGGCCACAAGTTCAGCGTGTCCGGCGAGGGCGAGGGCGATGCCACCTACGGCAAGCTGACCCTGAAGTTCATCTGCACCACCGGCAAGCTGCCCGTGCCCTGGCCCACCCTCGTGACCACCTTCACCTACGGCGTGCAGTGCTTCGCCCGCTACCCCGACCACATGAAGCAGCACGACTTCTTCAAGTCCGCCATGCCCGAAGGCTACGTCCAGGAGCGCACCATCTTCTTCAAGGACGACGGCAACTACAAGACCCGCGCCGAGGTGAAGTTCGAGGGCGACACCCTGGTGAACCGCATCGAGCTGAAGGGCATCGACTTCAAGGAGGACGGCAACATCCTGGGGCACAAGCTGGAGTACAACTACAACAGCCACAAGGTCTATATCACCGCCGACAAGCAGAAGAACGGCATCAAGGTGAACTTCAAGACCCGCCACAACATCGAGGACGGCAGCGTGCAGCTCGCCGACCACTACCAGCAGAACACCCCCATCGGCGACGGCCCCGTGCTGCTGCCCGACAACCACTACCTGAGCACCCAGTCCGCCCTGAGCAAAGACCCCAACGAGAAGCGCGATCACATGGTCCTGCTGGAGTTCGTGACCGCCGCCGGGATCACTCTCGGCATGGACGAGCTGTACAAG**TAATCTAGAGGGCCCGTTTAAACCCGCTGATCAGCCTCGATAAGATACATTGATGAGTTTGGACAAACCACAACTAGAATGCAGTGAAAAAAATGCTTTATTTGTGAAATTTGTGATGCTATTGCTTTATTTGTAACCATTATAAGCTGCAATAAACAAGTTGTCGAC**ACCCTGCCGTGTACCAGCTGAGAGACTCTAAATCCAGTGACAAGTCTGTCTGCCTATTCACCGATTTTGATTCTCAAACAAATGTGTCACAAAGTAAGGATTCTGATGTGTATATCACAGACAAAACTGTGCTAGACATGAGGTCTATGGACTTCAAGAGCAACAGTGCTGTGGCCTGGAGCAACAAATCTGACTTTGCATGTGCAAACGCCTTCAACAACAGCATTATTCCAGAAGACACCTTCTTCCC**GCTCTTCACAGCCCTCAGCTCTGATTTTGTAGGTAACCACGTGCGGACCGAGCGGCCGCAGGAACCCCTAGTGATGGAGTTGGCCACTCCCTCTCTGCGCGCTCGCTCGCTCACTGAGGCCGGGCGACCAAAGGTCGCCCGACGCCCGGGCTTTGCCCGGGCGGCCTCAGTGAGCGAGCGAGCGCGCAGCTGCCTGCAGGGGCGCCTGATGCGGTATTTTCTCCTTACGCATCTGTGCGGTATTTCACACCGCATACGTCAAAGCAACCATAGTACGCGCCCTGTAGCGGCGCATTAAGCGCGGCGGGTGTGGTGGTTACGCGCAGCGTGACCGCTACACTTGCCAGCGCCCTAGCGCCCGCTCCTTTCGCTTTCTTCCCTTCCTTTCTCGCCACGTTCGCCGGCTTTCCCCGTCAAGCTCTAAATCGGGGGCTCCCTTTAGGGTTCCGATTTAGTGCTTTACGGCACCTCGACCCCAAAAAACTTGATTTGGGTGATGGTTCACGTAGTGGGCCATCGCCCTGATAGACGGTTTTTCGCCCTTTGACGTTGGAGTCCACGTTCTTTAATAGTGGACTCTTGTTCCAAACTGGAACAACACTCAACCCTATCTCGGGCTATTCTTTTGATTTATAAGGGATTTTGCCGATTTCGGCCTATTGGTTAAAAAATGAGCTGATTTAACAAAAATTTAACGCGAATTTTAACAAAATATTAACGTTTACAATTTTATGGTGCACTCTCAGTACAATCTGCTCTGATGCCGCATAGTTAAGCCAGCCCCGACACCCGCCAACACCCGCTGACGCGCCCTGACGGGCTTGTCTGCTCCCGGCATCCGCTTACAGACAAGCTGTGACCGTCTCCGGGAGCTGCATGTGTCAGAGGTTTTCACCGTCATCACCGAAACGCGCGAGACGAAAGGGCCTCGTGATACGCCTATTTTTATAGGTTAATGTCATGATAATAATGGTTTCTTAGACGTCAGGTGGCACTTTTCGGGGAAATGTGCGCGGAACCCCTATTTGTTTATTTTTCTAAATACATTCAAATATGTATCCGCTCATGAGACAATAACCCTGATAAATGCTTCAATAATATTGAAAAAGGAAGAGT**ATGAGTATTCAACATTTCCGTGTCGCCCTTATTCCCTTTTTTGCGGCATTTTGCCTTCCTGTTTTTGCTCACCCAGAAACGCTGGTGAAAGTAAAAGATGCTGAAGATCAGTTGGGTGCACGAGTGGGTTACATCGAACTGGATCTCAACAGCGGTAAGATCCTTGAGAGTTTTCGCCCCGAAGAACGTTTTCCAATGATGAGCACTTTTAAAGTTCTGCTATGTGGCGCGGTATTATCCCGTATTGACGCCGGGCAAGAGCAACTCGGTCGCCGCATACACTATTCTCAGAATGACTTGGTTGAGTACTCACCAGTCACAGAAAAGCATCTTACGGATGGCATGACAGTAAGAGAATTATGCAGTGCTGCCATAACCATGAGTGATAACACTGCGGCCAACTTACTTCTGACAACGATCGGAGGACCGAAGGAGCTAACCGCTTTTTTGCACAACATGGGGGATCATGTAACTCGCCTTGATCGTTGGGAACCGGAGCTGAATGAAGCCATACCAAACGACGAGCGTGACACCACGATGCCTGTAGCAATGGCAACAACGTTGCGCAAACTATTAACTGGCGAACTACTTACTCTAGCTTCCCGGCAACAATTAATAGACTGGATGGAGGCGGATAAAGTTGCAGGACCACTTCTGCGCTCGGCCCTTCCGGCTGGCTGGTTTATTGCTGATAAATCTGGAGCCGGTGAGCGTGGGTCTCGCGGTATCATTGCAGCACTGGGGCCAGATGGTAAGCCCTCCCGTATCGTAGTTATCTACACGACGGGGAGTCAGGCAACTATGGATGAACGAAATAGACAGATCGCTGAGATAGGTGCCTCACTGATTAAGCATTGGTAA**CTGTCAGACCAAGTTTACTCATATATACTTTAGATTGATTTAAAACTTCATTTTTAATTTAAAAGGATCTAGGTGAAGATCCTTTTTGATAATCTCATGACCAAAATCCCTTAACGTGAGTTTTCGTTCCACTGAGCGTCAGACCCCGTAGAAAAGATCAAAGGATCTTCTTGAGATCCTTTTTTTCTGCGCGTAATCTGCTGCTTGCAAACAAAAAAACCACCGCTACCAGCGGTGGTTTGTTTGCCGGATCAAGAGCTACCAACTCTTTTTCCGAAGGTAACTGGCTTCAGCAGAGCGCAGATACCAAATACTGTCCTTCTAGTGTAGCCGTAGTTAGGCCACCACTTCAAGAACTCTGTAGCACCGCCTACATACCTCGCTCTGCTAATCCTGTTACCAGTGGCTGCTGCCAGTGGCGATAAGTCGTGTCTTACCGGGTTGGACTCAAGACGATAGTTACCGGATAAGGCGCAGCGGTCGGGCTGAACGGGGGGTTCGTGCACACAGCCCAGCTTGGAGCGAACGACCTACACCGAACTGAGATACCTACAGCGTGAGCTATGAGAAAGCGCCACGCTTCCCGAAGGGAGAAAGGCGGACAGGTATCCGGTAAGCGGCAGGGTCGGAACAGGAGAGCGCACGAGGGAGCTTCCAGGGGGAAACGCCTGGTATCTTTATAGTCCTGTCGGGTTTCGCCACCTCTGACTTGAGCGTCGATTTTTGTGATGCTCGTCAGGGGGGCGGAGCCTATGGAAAAACGCCAGCAACGCGGCCTTTTTACGGTTCCTGGCCTTTTGCTGGCCTTTTGCTCACATGT

**Supplementary Note 2.** README, instructions how to use provided codes.

The scripts were written and tested on Linux - no reason to think it can't work on any OS

The following software is needed:

* CRISPresso2 - tested using version 2.1.1 - see https://github.com/pinellolab/CRISPResso2

* R - tested with version 3.4.2 with the addition of the xlsx package - see https://cran.r-project.org/web/packages/xlsx/xlsx.pdf for details.

* Python - tested on version 3.6.6

The time taken to run the conversion scripts is dependent on how many samples/amplicons are in the CRISPResso run.

Typically, the runtime is not an issue.

Briefly the process used in analysis of the samples is:

1. Create the amplicons file ($amplicons_file) with at least these 3 tab-separated columns:

SiteName ReferenceSequence GuideSequence

The GuideSequence should exclude the PAM, which is assumed to be at the 3' end.

The GuideSequence should also be a sub-sequence of the ReferenceSequence.

2. Run CRISPRessoPooled:

CRISPRessoPooled -r1 R1.fastq.gz -r2 R2.fastq.gz -f $amplicons_file -n $name -o $crispresso2_dir ...

See https://github.com/pinellolab/CRISPResso2#crispressopooled for details

3. Convert the AlleleFrequecy.zip files generated by CRISPRessoPooled to samples and variant txt files, e.g.:

crispresso2rima.py --amplicons $amplicons_file --window 8 --out $output_dir $crispresso2_dir/$name

This is a python3 script using standard modules - run without arguments to see full usage.

4. Convert variant txt files to xlsx and insert samples and paths/URLs into the RIMA template xlsm, e.g.:

Rscript ./make_rima.R \

RIMA3_Template.xlsm \

$output_dir/RIMA-samples.txt \

$output_dir \

$output_dir

This R script requires the xlsx library - see https://cran.r-project.org/web/packages/xlsx/xlsx.pdf for details.

The arguments are:

Path to the template xlsm file (supplied)

Path to generated samples.txt file

Path to the output directory for xlsx and xlsm files

Path/URL to insert into the template for Excel to locate the xlsx files

5. Launch Excel on the $output_dir/RIMA.xlsm and enable macros.
