# Supplementary material for: Simultaneous inhibition of DNA-PK and Polϴ improves integration efficiency and precision of genome editing
Source: Nat Commun. 2023 Aug 14;14:4761. doi: 10.1038/s41467-023-40344-4 (PMC10425386; doi:10.1038/s41467-023-40344-4)
Supplement: Supplementary file 2 — Reporting Summary [file 41467_2023_40344_MOESM2_ESM.pdf]

nature portfolio

Corresponding author(s): Sandra Wimberger
Marcello Maresca

Last updated by author(s): Jul 21, 2023

Reporting Summary

Nature Portfolio wishes to improve the reproducibility of the work that we publish. This form provides structure for consistency and transparency in reporting. For further information on Nature Portfolio policies, see our Editorial Policies and the Editorial Policy Checklist.

Statistics

For all statistical analyses, confirm that the following items are present in the figure legend, table legend, main text, or Methods section.

n/a	Confirmed	
☐	☒	The exact sample size (*n*) for each experimental group/condition, given as a discrete number and unit of measurement
☐	☒	A statement on whether measurements were taken from distinct samples or whether the same sample was measured repeatedly
☐	☒	The statistical test(s) used AND whether they are one- or two-sided<br>*Only common tests should be described solely by name; describe more complex techniques in the Methods section.*
☒	☐	A description of all covariates tested
☒	☐	A description of any assumptions or corrections, such as tests of normality and adjustment for multiple comparisons
☐	☒	A full description of the statistical parameters including central tendency (e.g. means) or other basic estimates (e.g. regression coefficient) AND variation (e.g. standard deviation) or associated estimates of uncertainty (e.g. confidence intervals)
☐	☒	For null hypothesis testing, the test statistic (e.g. *F*, *t*, *r*) with confidence intervals, effect sizes, degrees of freedom and *P* value noted<br>*Give P values as exact values whenever suitable.*
☒	☐	For Bayesian analysis, information on the choice of priors and Markov chain Monte Carlo settings
☒	☐	For hierarchical and complex designs, identification of the appropriate level for tests and full reporting of outcomes
☒	☐	Estimates of effect sizes (e.g. Cohen's *d*, Pearson's *r*), indicating how they were calculated

Our web collection on statistics for biologists contains articles on many of the points above.

Software and code

Policy information about availability of computer code

Data collection

No code was used to collect the data.

Data analysis

Statistical analysis and data visualization: Graphpad Prism 8.4.3 (GraphPad Software, Inc), JMP 16.1.0 (SAS Institute Inc.)
ddPCR: QuantaSoft 1.7.4 (Bio-Rad)
Flow cytometer: ForeCyt® Enterprise Client Edition 6.2 and FlowJo v10.8.0
NGS analysis:
Amplicon-Sequencing:
De-multiplexing - Bcl2Fastq software version 2.20.0.422 (Illumina)
Analysis: CRISPResso version 2.1.1 (PMID: 30809026), RIMA (PMID: 30032200), Python3, R version 3.4.2
The RIMA v2 file is available in the supplementary macro-enabled Excel file. Supplementary Code 1 contains a Python script to process CRISPResso2 runs into .txt files for RIMA v2. Supplementary Code2 comprises an R script to convert .txt files to .xlsx files and adds file paths to the RIMA v2 template. Supplementary Note 2 describes how to use the different scripts. The code is freely available under the MIT license. All files are accessible in the Supplementary Software zipped folder.

PacBio long-read sequencing:
PacBio ccs version 6.4.0; pbmm2 version 1.9.0 and minimap2 version 2.15 (PMID: 29750242), bedtools version 2.20.0 (PMID: 20110278), deeptools version 3.5.1 (PMID: 24799436),
samtools version 1.15.1, JBrowse 2 version 1.7.6 (PMID: 27072794)

March 2021

For manuscripts utilizing custom algorithms or software that are central to the research but not yet described in published literature, software must be made available to editors and reviewers. We strongly encourage code deposition in a community repository (e.g. GitHub). See the Nature Portfolio guidelines for submitting code & software for further information.

Data

Policy information about availability of data

All manuscripts must include a data availability statement. This statement should provide the following information, where applicable:

- Accession codes, unique identifiers, or web links for publicly available datasets
- A description of any restrictions on data availability
- For clinical datasets or third party data, please ensure that the statement adheres to our policy

Next generation sequencing data are available in the NCBI Sequence Read Archive database (SRA) under BioProject accession code PRJNA907774 [https://www.ncbi.nlm.nih.gov/bioproject/PRJNA907774/]. Source data are provided with this paper.

Human research participants

Policy information about studies involving human research participants and Sex and Gender in Research.

Reporting on sex and gender	primary human CD4+ T cells: 2 males, 1 female, based on self-report. primary human CD3+ T cells: N/A – NHS-BT do not provide information on gender of their blood donors. Sex and/or gender was not considered in the study design.
Population characteristics	primary human CD4+ T cells: due to the risk to identify the subjects, this information was not provided. primary human CD3+ T cells: Healthy donors sourced in the UK as part of the NHS-BT programme.
Recruitment	primary human CD4+ T cells: Healthy donors were recruited from AstraZeneca volunteers and all samples were taken following appropriate blood collection guidelines. primary human CD3+ T cells: Donor recruitment managed by NHS-BT programme.
Ethics oversight	primary human CD4+ T cells: all blood donor volunteers signed Informed Consent form and donation was approved by AstraZeneca's Institutional review board and local Ethic committee (033-10).<br><br>primary human CD3+ T cells: human peripheral blood mononuclear cells (PBMCs) were isolated from leukocyte cones (supplied by NHS Blood and Transplant Service (NHSBT, UK). This report is independent research. NHS Blood & Transplant have provided material in support of the research. The views expressed in this publication are those of the author(s) and not necessarily those of NHS Blood & Transplant. AstraZeneca has a governance framework and processes in place to ensure that commercial sources have appropriate patient consent and ethical approval in place for collection of the samples for research purposes including use by for-profit companies. The AstraZeneca Biobank in the UK is licensed by the Human Tissue Authority (Licence No. 12109) and has National Research Ethics Service Committee (NREC) Approval as a Research Tissue Bank (RTB) (REC No 22/NW/0102) which covers the use of the samples for this project.

Note that full information on the approval of the study protocol must also be provided in the manuscript.

Field-specific reporting

Please select the one below that is the best fit for your research. If you are not sure, read the appropriate sections before making your selection.

[x] Life sciences [ ] Behavioural & social sciences [ ] Ecological, evolutionary & environmental sciences

For a reference copy of the document with all sections, see nature.com/documents/nr-reporting-summary-flat.pdf

Life sciences study design

All studies must disclose on these points even when the disclosure is negative.

Sample size	Sample sizes were determined based on literature precedence for genome editing experiments (e.g. Peterka et al., 2022 Nat. Comm.)
Data exclusions	Figure 2c ssDNA, AZD7648 n=2<br>Figure 2d ssDNA, AZD7648 1.25 uM n=2<br>Figure 2e, AZD7648 uM n=2<br>Supplementary Figure S4b, AZD7648 1.25 uM n=2; AURBK 5 uM n=2, Fen1 5 uM n=2<br>Figure 4d AZD7648 + POLQ n=2<br>Supplementary Figure S4b, AZD7648 1.25 uM n=2; AURBK 5 uM n=2; Fen1 5 uM n=2<br>Figure 5a, HEK3 OT1 ssDNA AZD7648, AZD7648+PolQi2 n=2

Replication	All experiment were performed with cells separated into three stocks, each individually transfected with same transfection mix, recovered, and analysed (technical replicate); or cells from separately cultured stocks were individually transfected with different transfection mix, recovered and analysed (biological replicate), as specified in figure legends.
Randomization	Mammalian cells were cultured under identical conditions, no randomization was used.
Blinding	Mammalian cells used in this study were grown under identical conditions, no blinding was used.

Reporting for specific materials, systems and methods

We require information from authors about some types of materials, experimental systems and methods used in many studies. Here, indicate whether each material, system or method listed is relevant to your study. If you are not sure if a list item applies to your research, read the appropriate section before selecting a response.

Materials & experimental systems

n/a	Involved in the study
☒	☐ Antibodies
☐	☒ Eukaryotic cell lines
☒	☐ Palaeontology and archaeology
☒	☐ Animals and other organisms
☒	☐ Clinical data
☒	☐ Dual use research of concern

Methods

n/a	Involved in the study
☒	☐ ChIP-seq
☐	☒ Flow cytometry
☒	☐ MRI-based neuroimaging

Eukaryotic cell lines

Policy information about cell lines and Sex and Gender in Research

Cell line source(s)	TLR parental clone HEK-293, ATCC CRL-1573<br>HEK293T, Genhunter corporation Q401<br>HepG2, ATCC HB-8065<br>Jurkat, DKMZ ACC-282, or American Type Culture Collection TIB-152<br>hiPSC line, R-iPSC Clone J, LineID: iPS.1<br>Primary human CD4+ T cells were derived from blood of healthy donors recruited from the AstraZeneca voluntary blood donation program<br>Cryopreserved PHH were derived from BioIVT F00995-P Lot.: VNL<br>Primary human CD3+ T cells were isolated from a healthy donor Leukopaks from NHS Blood and Transplant Service (NHS-BT UK)<br>Detailed descriptions are available in the methods section.
Authentication	Cell line identities were confirmed through STR profiling.
Mycoplasma contamination	Cell lines were regularly tested for mycoplasma contaminations. All cells tested negative.
Commonly misidentified lines (See ICLAC register)	No commonly misidentified lines were used.

Flow Cytometry

Plots

Confirm that:

☒ The axis labels state the marker and fluorochrome used (e.g. CD4-FITC).

☒ The axis scales are clearly visible. Include numbers along axes only for bottom left plot of group (a 'group' is an analysis of identical markers).

☒ All plots are contour plots with outliers or pseudocolor plots.

☒ A numerical value for number of cells or percentage (with statistics) is provided.

Methodology

Sample preparation	TLR screen: Medium was removed and cells were detached with Trypsin-EDTA (0.25%) (Gibco). FACS buffer (PBS (pH 7.4 (Gibco)) 1 mM EDTA (Ambion), 2% (v/v) FBS (Gibco)) and FL-4 well identification beads (AH Diagnositc) were added to the wells and cells were transferred to 384-well round bottom plates (Corning).

	Primary human CD3+ cells: ~1.5 x 10^5 cells were pelleted (300 x g for 5 min). Cells were washed once in FACS Buffer (PBS 1 % BSA) and resuspended in 300 ul FACS buffer.
Instrument	TLR screen samples were analysed with Intellicyte iQue screener (Sartorius).<br>Primary human T cells were analysed with BD LSR-Fortessa Cell Analyzer (BD Biosciences).
Software	ForeCyt® Enterprise Client Edition 6.2<br>FlowJo v10.8.0
Cell population abundance	Samples eligible for the screen contained more than 1,000 cells per well. Percent values to generate the figures are provided in the Source Data files.<br>Primary human T cells : Percentages of populations relevant to this analysis (lymphocytes/ single cells/ GFP+) were determined as per gating strategy below. Live cell counts of knock-in containing cells (KI+) are illustrated in Supplementary Figure S10d. Percent values to generate the figures are provided in the Source data files.
Gating strategy	Gating strategy for TLR screen is based on forward scatter (FSC) and side scatter (SSC), subsequently gated for singlets with FSC height (FSC-H) and FSC-area (FSC-A). Followed by eGFP and dsRed quantification in the single cell population. Gates were defined based on control samples as follows; DsRed-positive cells >15% as an increase of NHEJ and DsRed-positive cells <8% as decrease of NHEJ. GFP-positive cells >4.5% were considered as an increase of HDR events. Compounds were regarded as inactive for DsRed-positive cells <15% and GFP-positive cells <4.5%. Gating strategy is exemplified in Supplementary Figure S1b.<br>Primary human T cells: (1) Lymphocyte gate (2) Doublet exclusion (3) % GFP as measure of KI efficiency (TRAC/ GFP KI) .<br>Gating strategy is exemplified in Supplementary Figure S1b.

☒ Tick this box to confirm that a figure exemplifying the gating strategy is provided in the Supplementary Information.
