# Supplementary material for: Simultaneous inhibition of DNA-PK and Polϴ improves integration efficiency and precision of genome editing
Source: Nat Commun. 2023 Aug 14;14:4761. doi: 10.1038/s41467-023-40344-4 (PMC10425386; doi:10.1038/s41467-023-40344-4)
Supplement: Supplementary file 4 — Description of Additional Supplementary Files [file 41467_2023_40344_MOESM4_ESM.pdf]

Title: Supplementary Data 1
Description: Summary of spacer sequences used in this study.

Title: Supplementary Data 2
Description: Summary of primers and probes used in this study.

Title: Supplementary Data 3
Description: Summary of DNA donor sequences used in this study.

Title: Supplementary Data 4
Description: Reference sequences for bioinformatic analysis used in this study.

Title: Supplementary Data 5
Description: Targets and pXC_{50} values for compounds used in the TLR screen, where target annotations are available.

Title: Supplementary Software
Description: Folder containing codes and instruction for deep targeted amplicon knock-in sequencing data analysis.

> **Title:** Supplementary Code 1.
> **Description:** Python Script to process CRISPResso2 runs into .txt files for RIMA.
>
> **Title:** Supplementary Code2.
> **Description:** R script to convert .txt files to .xlsx files and add paths into RIMA template.
>
> **Title:** RIMAv2 Template & How to run RIMA2.
> **Description:** RIMA v2 macro-enabled Excel file and instructions.
>
> **Title:** Supplementary Note S2.
> **Description:** README – User instructions for provided code.
