# Supplementary material for: Simultaneous inhibition of DNA-PK and Polϴ improves integration efficiency and precision of genome editing
Source: Nat Commun. 2023 Aug 14;14:4761. doi: 10.1038/s41467-023-40344-4 (PMC10425386; doi:10.1038/s41467-023-40344-4)
Supplement: Supplementary file 10 — Supplementary Software [file 41467_2023_40344_MOESM10_ESM.zip › Supplementary Note 2.docx]

**Supplementary Note 2.** README, instructions how to use provided codes.

The scripts were written and tested on Linux - no reason to think it can't work on any OS

The following software is needed:

* CRISPresso2 - tested using version 2.1.1 - see https://github.com/pinellolab/CRISPResso2

* R - tested with version 3.4.2 with the addition of the xlsx package - see https://cran.r-project.org/web/packages/xlsx/xlsx.pdf for details.

* Python - tested on version 3.6.6

The time taken to run the conversion scripts is dependent on how many samples/amplicons are in the CRISPResso run.

Typically, the runtime is not an issue.

Briefly the process used in analysis of the samples is:

1. Create the amplicons file ($amplicons_file) with at least these 3 tab-separated columns:

SiteName ReferenceSequence GuideSequence

The GuideSequence should exclude the PAM, which is assumed to be at the 3' end.

The GuideSequence should also be a sub-sequence of the ReferenceSequence.

2. Run CRISPRessoPooled:

CRISPRessoPooled -r1 R1.fastq.gz -r2 R2.fastq.gz -f $amplicons_file -n $name -o $crispresso2_dir ...

See https://github.com/pinellolab/CRISPResso2#crispressopooled for details

3. Convert the AlleleFrequecy.zip files generated by CRISPRessoPooled to samples and variant txt files, e.g.:

crispresso2rima.py --amplicons $amplicons_file --window 8 --out $output_dir $crispresso2_dir/$name

This is a python3 script using standard modules - run without arguments to see full usage.

4. Convert variant txt files to xlsx and insert samples and paths/URLs into the RIMA template xlsm, e.g.:

Rscript ./make_rima.R \

RIMA3_Template.xlsm \

$output_dir/RIMA-samples.txt \

$output_dir \

$output_dir

This R script requires the xlsx library - see https://cran.r-project.org/web/packages/xlsx/xlsx.pdf for details.

The arguments are:

Path to the template xlsm file (supplied)

Path to generated samples.txt file

Path to the output directory for xlsx and xlsm files

Path/URL to insert into the template for Excel to locate the xlsx files

5. Launch Excel on the $output_dir/RIMA.xlsm and enable macros.
